# Supplementary figures and images for: Targeting the ubiquitin‐proteasome system in a pancreatic cancer subtype with hyperactive MYC
Source: Mol Oncol. 2020 Nov 8;14(12):3048–64. doi: 10.1002/1878-0261.12835 (PMC7718946; doi:10.1002/1878-0261.12835)

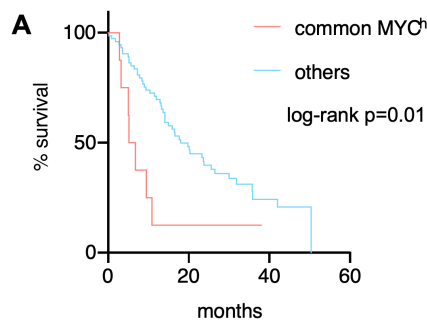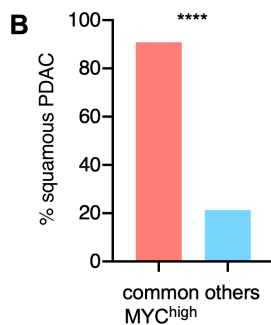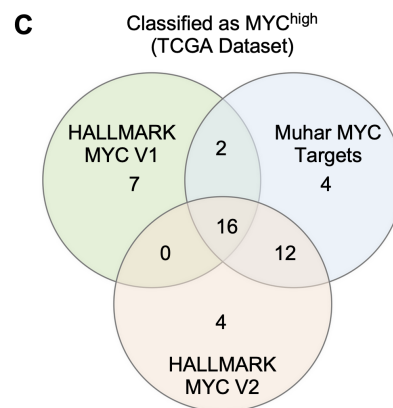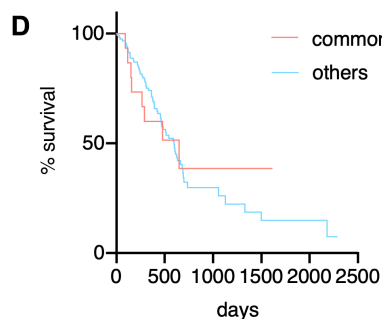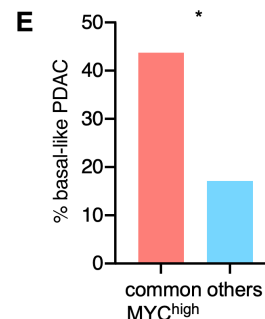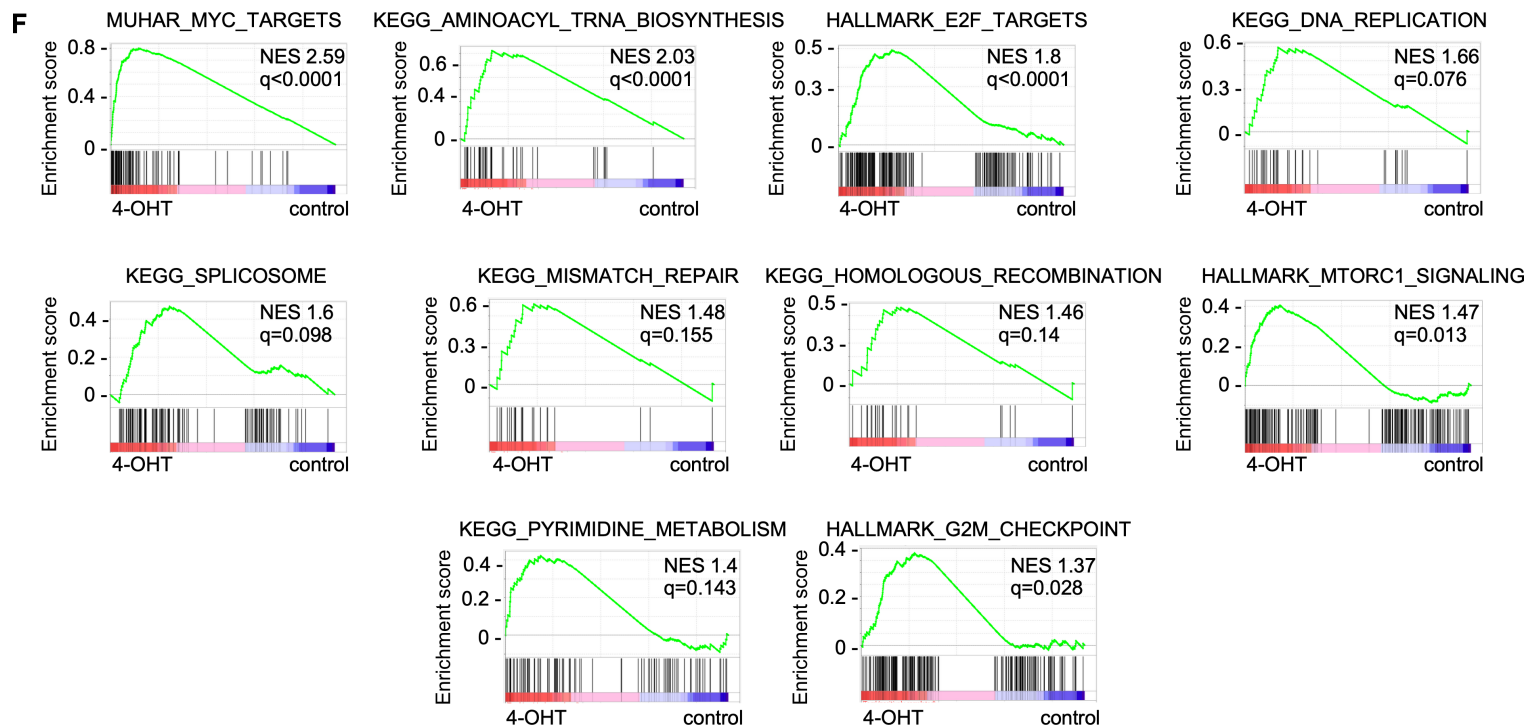

Supplement: Supplementary file 1 — Fig. S1. Survival and Subtypes of common MYChigh PDACs. A) Survival data of common MYChigh PDACs of the ICGC dataset are displayed in a Kaplan–Meier curve. P value of a log‐rank test is depicted. B) Percentage of squamous subtype of the common MYChigh PDACs compared to the others. Fisher Exact test: P < 0.0001. C) Venn diagram of PDAC identified as MYChigh by clustering of the genes of the depicted signatures in the TCGA dataset. 16 PDACs were identified as common MYChigh PDACs. D) Survival data of common MYChigh PDACs of the TCGA dataset are displayed in a Kaplan–Meier curve. E) Percentage of basal‐like subtype of the common MYChigh PDACs compared to the others. Fisher Exact test: P < 0.05. F) GSEA of IMIM‐PC1MYCER cells treated with 4‐OHT to activate MYC. Depicted are HALLMARK and KEGG signature corresponding to the tissue‐based analysis corresponding to Fig. 3D. The NES and the FDR q values are depicted. [file MOL2-14-3048-s001.pdf]

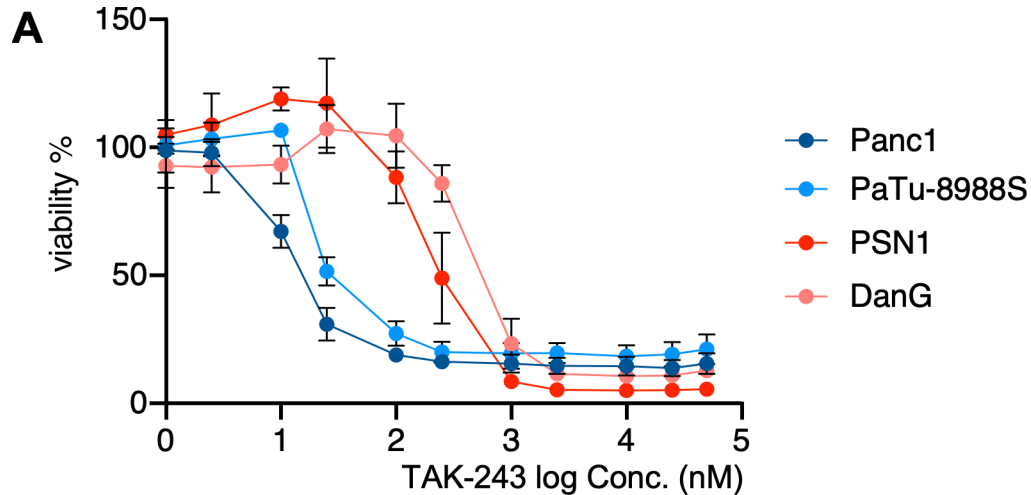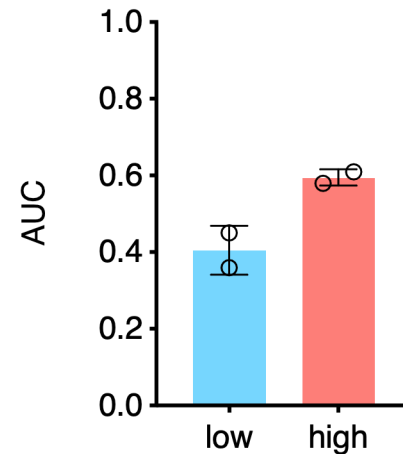

**B**

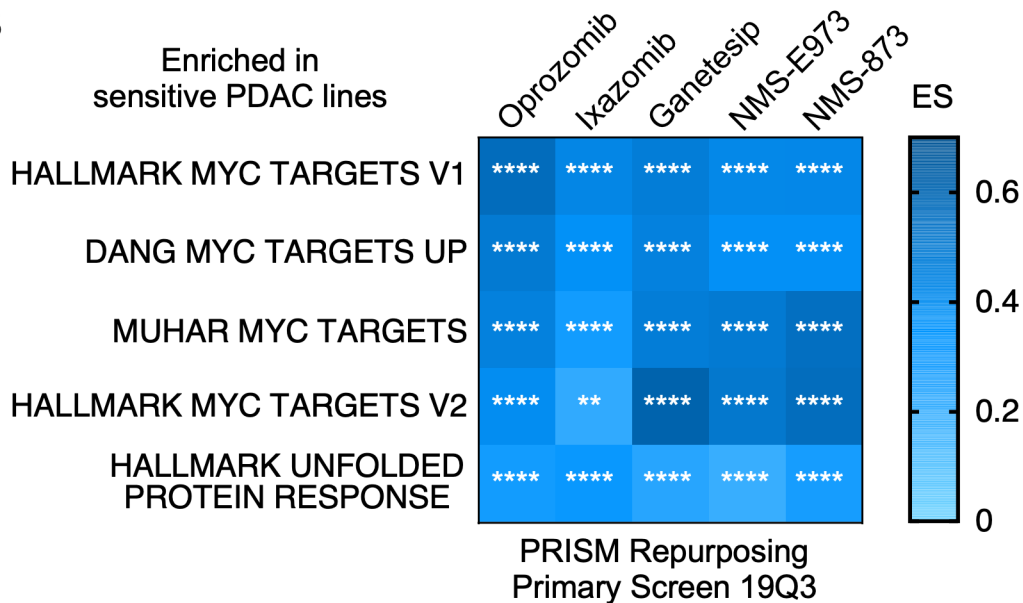

Supplement: Supplementary file 2 — Fig. S2. Association of MYC with perturbants of the protein homeostasis. Sensitivities of human PDAC cell lines from the PRISM repurposing primary screen (19Q3) [30] of the depicted drug classes were divided into quartiles and lines for the most sensitive quartile were compared to the remaining cell lines of the complete CCLE‐PDAC dataset with a gene set enrichment analysis using the GeneTrail2 1.6 web service. The enrichment score was color‐coded. ** adjust. P‐value < 0.01; **** adjust. P‐value < 0.0001. [file MOL2-14-3048-s002.pdf]
