# Supplementary material for: Targeting the ubiquitin‐proteasome system in a pancreatic cancer subtype with hyperactive MYC
Source: Mol Oncol. 2020 Nov 8;14(12):3048–64. doi: 10.1002/1878-0261.12835 (PMC7718946; doi:10.1002/1878-0261.12835)
Supplement: Supplementary file 3 — Table S1. Doubling time of PDAC cell lines used for the drug screening experiment. Panc1, PaTu8988S, DanG and PSN1 cells were seeded in 96 well plates at a density of 3000 cells/well. After 24, 48, 72 and 96 h viable cells were measured by MTT test to determine doubling time of the cell lines. [file MOL2-14-3048-s003.pdf]

cell line ID	doubling time (h)
Panc1	44.78
Patu8988S	48.86
DanG	45.62
PSN1	27.31
