# Supplementary material for: Targeting the ubiquitin‐proteasome system in a pancreatic cancer subtype with hyperactive MYC
Source: Mol Oncol. 2020 Nov 8;14(12):3048–64. doi: 10.1002/1878-0261.12835 (PMC7718946; doi:10.1002/1878-0261.12835)
Supplement: Supplementary file 4 — Table S2. Complete list of the response to compounds used in the drug screen. PSN1, Panc1, PaTu8988S and DanG cells were treated with n = 129 compounds in biological and technical triplicates. Drug response as well as mean of MYC high and mean of MYC low are displayed. [file MOL2-14-3048-s004.pdf]

	Panc1 MYC^{low}	Patu8988S MYC^{low}	DanG MYC^{high}	PSN1 MYC^{high}	Mean MYC^{high} / Mean MYC^{low}
Panobinostat	0.5927368	0.4316097	0.1283566	0.1012438	4.461432123
Cladribine	1.0362948	0.8812915	0.3784469	0.1964837	3.335335254
Idarubicin hydrochloride	0.3853088	0.3580649	0.1316619	0.106136	3.126073667
Clofarabine	0.6907533	0.9303737	0.3631497	0.1735106	3.020768958
Topotecan hydrochloride	0.6338552	0.7889655	0.3766401	0.146818	2.718118017
Gemcitabine hydrochloride	0.6285325	0.7472625	0.3121124	0.2189043	2.590869276
Floxuridine	0.7672699	0.9213013	0.489905	0.3033217	2.128737275
Doxorubicin hydrochloride	0.5381085	0.4160912	0.309046	0.1406154	2.122040469
Bortezomib	0.1740942	0.3174964	0.132343	0.1032272	2.086811521
Dactinomycin	0.2778748	0.4070966	0.2398357	0.0918611	2.065052824
Pralatrexate	0.5334845	0.8566843	0.3301672	0.3439406	2.062235104
Daunorubicin hydrochloride	0.3201861	0.6879631	0.4281178	0.084397	1.967063898
Dasatinib	1.0649763	0.8051341	0.3248287	0.6516466	1.915164076
Methotrexate	0.543753	0.8269723	0.3974606	0.3274105	1.890991486
Romidepsin	0.6142233	0.6397273	0.4472008	0.2266042	1.860999368
Pemetrexed, Disodium salt, Heptahydrate	1.1160514	0.8835727	0.7531286	0.3413614	1.826991624
Ixabepilone	0.6076967	0.4359349	0.1981229	0.3851835	1.789165199
Epirubicin hydrochloride	0.5976475	0.600278	0.4432164	0.2435132	1.744391794
Paclitaxel	0.5937123	0.4850653	0.2299463	0.3899297	1.740312281
Mitomycin	0.8186603	0.7457391	0.5472753	0.3686277	1.708040398
Trametinib	1.0522746	0.3858011	0.5152489	0.3443984	1.672867182
Belinostat	1.2235609	0.9651008	0.6105508	0.742512	1.617561069
Plicamycin	0.2406453	0.5661695	0.3741679	0.1455876	1.552296875
Docetaxel	0.6006985	0.358746	0.203751	0.4255217	1.524688004
Cytarabine hydrochloride	0.7906227	0.7922291	0.4905566	0.5891065	1.466060789
Valrubicin	0.658399	0.6932424	0.6135694	0.3178223	1.4512062
Vincristine sulfate	0.4842991	0.4904168	0.3563123	0.3683089	1.34513844
Cobimetinib	0.8057013	0.4227304	0.5749653	0.3467257	1.332801963
Cabazitaxel	0.5594477	0.4260106	0.3483661	0.3990782	1.318437226
Vinblastine sulfate	0.4661904	0.6971546	0.5479773	0.3792634	1.254630907
Teniposide	0.7099909	0.6541106	0.5130224	0.5785205	1.249700352
Vorinostat	1.1984255	1.0365938	0.8138551	0.985923	1.241830561
Mitoxantrone	0.4111703	0.360539	0.4180332	0.2049134	1.23880501
Carfilzomib	0.123085	0.2872714	0.1195335	0.2173406	1.218130104
Pazopanib hydrochloride	1.1507001	1.1699473	0.9319465	0.9738086	1.217704961
Tamoxifen citrate	1.2258223	0.8767898	0.8112547	0.9373592	1.202445098
Vemurafenib	1.2983508	1.0166108	0.9096793	1.0162834	1.201976341
Bleomycin sulfate	0.9200904	0.8969911	0.7066424	0.8128505	1.195847316
Axitinib	1.0551648	1.0017045	0.816527	0.9102405	1.191167527
Thioguanine	1.0981832	0.8629344	1.0158377	0.6383698	1.185533008
Vinorelbine tartrate	0.5201178	0.5633463	0.497125	0.4253102	1.174569397
Sorafenib	1.162171	1.2830505	1.0178113	1.0745961	1.168616375
Lenvatinib	1.149886	1.0538571	0.8996172	0.9908693	1.165701528
Ponatinib	0.9676809	0.8053608	0.730816	0.8036482	1.155479361
Trifluridine	1.1512183	1.0878322	0.9053789	1.0408002	1.150485363
Pomalidomide	1.0906291	1.0283482	0.9547646	0.9212327	1.129520454
Celecoxib	1.1502735	1.1248195	0.9611498	1.0822925	1.113362995
Idelalisib	1.1145138	1.0326372	0.9856933	0.9471336	1.110886312
Anastrozole	1.0940645	1.0583861	0.9622566	0.9757917	1.110627962
Megestrol acetate	1.1106453	1.1060051	0.9991441	1.0044367	1.106344418
Mechlorethamine hydrochloride	1.1069868	0.9741389	1.0350162	0.8476075	1.105438979
Raloxifene	1.0973425	1.055331	1.0202812	0.9277671	1.1050411
Chlorambucil	1.2870682	1.0877594	1.0875412	1.0623749	1.104614114
Omacetaxine mepesuccinate	0.1392018	0.5614243	0.4482203	0.1877929	1.101590466
Carboplatin	1.1176001	1.0182414	0.9936223	0.9558329	1.095609456

Regorafenib	1.1063028	0.9268283	0.9606536	0.8963906	1.094821059
Decitabine	0.9667371	0.9534292	0.622724	1.1400811	1.089267518
Afatinib	1.0705093	0.892516	0.8782874	0.9339288	1.083218081
Hydroxyurea	1.1290124	0.9838368	0.9961748	0.9549181	1.082905492
Amifostine	1.0944602	1.0792695	0.9797668	1.0289677	1.082138826
Fluorouracil	1.0876493	1.0280319	0.9256822	1.0320756	1.08066543
Mitotane	1.1561005	1.0283327	1.0450807	0.9784554	1.079512906
Crizotinib	1.009358	0.8524901	0.7347524	0.9914502	1.078580271
Carmustine	1.1694594	1.0111272	1.0154788	1.0152511	1.073794532
Lenalidomide	1.1758563	1.0865372	0.9625862	1.1516524	1.070074789
Arsenic trioxide	1.1543637	1.0026565	1.0099186	1.0098961	1.067929769
Dabrafenib mesylate	1.0476995	0.7038174	0.6271765	1.0143573	1.067000238
Streptozocin	1.0629211	1.1350681	1.0200487	1.0443164	1.064728879
Estramustine phosphate sodium	1.1174815	0.9156263	0.9230253	0.9915053	1.061935398
Aminolevulinic acid hydrochloride	1.2735396	1.0125003	1.0472284	1.1059952	1.061682585
Vismodegib	1.1140285	1.0051374	0.978548	1.0176179	1.061618157
Methoxsalen	1.1077994	1.0322965	1.0017812	1.017235	1.059969685
Uridine triacetate	1.1105191	0.934566	0.9040666	1.026801	1.05915346
Ifosfamide	1.0673416	1.0517827	0.9984629	1.0063229	1.057032755
Pentostatin	1.1168985	1.1328357	1.0000841	1.1287894	1.056772122
Etoposide	0.8257375	0.9160592	0.8008493	0.8583683	1.049769979
Plerixafor	1.0156081	1.0299569	0.9693547	0.9840821	1.047162072
Mercaptopurine	1.1533893	1.064621	1.0421167	1.0762546	1.047035651
Temozolomide	1.100926	0.9987936	1.0170777	0.9943394	1.043900587
Melphalan hydrochloride	1.1167922	0.8871951	0.9884728	0.9317933	1.043598772
Gefitinib	1.0671326	0.9157831	0.9355389	0.9649645	1.043363337
Busulfan	1.1515438	0.9183881	1.019652	0.9703823	1.040148863
Exemestane	1.0891907	1.0271264	1.0561781	0.9864502	1.036075461
Altretamine	1.2215769	0.9396757	1.0638617	1.0242761	1.035014345
Triethylenemelamine	0.8480403	0.8116295	0.6936217	0.9105794	1.034577136
Ibrutinib	1.0167125	0.8967658	0.9005192	0.9503861	1.033806651
Temsirolimus	0.7991547	0.7438944	0.5569734	0.9390997	1.031399575
Pipobroman	1.0421908	1.0351135	0.9131288	1.1086645	1.027456309
Cyclophosphamide	1.0963373	1.0779427	1.0257545	1.0921417	1.026622568
Nelarabine	1.0364137	1.0449675	0.9784018	1.0608611	1.020653647
Dacarbazine	1.0773743	0.9799451	0.9998566	1.0329581	1.012054519
Lapatinib	1.2118227	1.0117246	1.0453586	1.1529822	1.011466159
Olaparib	1.0333574	0.9618045	0.9874746	0.9953946	1.006199381
Azacitidine	1.1057188	0.8669931	0.8983152	1.0624277	1.006104312
Sunitinib	1.125673	0.9359795	1.0082527	1.0437547	1.004700285
Procarbazine hydrochloride	1.0467224	0.9620981	0.9780714	1.0236254	1.003558771
Cisplatin	0.9783246	0.9864485	0.9717884	0.9881982	1.002442154
Thalidomide	1.1016887	0.8760751	0.9574126	1.0155748	1.002420886
Capecitabine	1.1033576	0.9785461	0.991254	1.087819	1.001361478
Fludarabine phosphate	1.0735069	0.9572477	1.0175318	1.0113273	1.000934311
Letrozole	0.964071	1.0074587	0.9956723	0.9810257	0.997385394
Ixazomib citrate	0.245811	0.5349317	0.3633141	0.4205316	0.996041413
Erismodegib	1.101577	0.8690601	0.9034578	1.0784045	0.99433599
Erlotinib hydrochloride	1.0831351	0.9835468	1.0028445	1.0771681	0.993591089
Imatinib	1.0910148	0.9407156	1.0124131	1.0327366	0.993438465
Irinotecan hydrochloride	1.0565756	0.8007072	0.9332201	0.9427394	0.990044197
Tretinoin	1.0659355	0.9976059	1.0807142	1.0058703	0.988956574
Dexrazoxane	1.0527764	0.9693531	0.9693255	1.0795052	0.986967628
Uracil mustard	1.001703	1.0043368	1.0454801	0.9906094	0.98524151
Allopurinol	1.1122121	1.0559206	1.0270413	1.1792553	0.982702291
Vandetanib	1.0904952	0.819051	0.9319007	1.0114376	0.98261128
Bendamustine hydrochloride	1.1159631	0.9338939	1.078548	1.0149397	0.979158848

Sirolimus	0.787637	0.736924	0.624014	0.9441733	0.972180429
Enzalutamide	0.9712496	0.9460363	1.008043	0.9676428	0.970440678
Nilotinib	1.1224254	1.146312	1.2644867	1.085551	0.965404678
Thiotepa	0.9899792	0.9029347	0.9891108	0.9768673	0.962835683
Everolimus	0.8114723	0.776071	0.6802286	0.9714906	0.961145951
Lomustine	0.9521525	0.9542411	1.0056073	0.9788209	0.960676527
Oxaliplatin	1.0346028	0.8738007	1.006481	0.9801285	0.960633421
Cabozantinib	1.0326035	0.8079131	0.8756074	1.0459597	0.95782062
Palbociclib	0.8332184	0.7235895	0.7367916	0.8895787	0.957228421
Fulvestrant	1.0301407	0.8667615	1.0025988	0.9857989	0.953985335
Zoledronic acid	1.1484752	0.9349664	1.1061776	1.0915123	0.94801437
Osimertinib	1.0508892	0.7746164	0.9351702	0.997295	0.94465117
Alectinib	0.9845287	0.765287	0.8784731	0.9897483	0.936621134
Ceritinib	0.9920572	0.6634162	0.9385272	0.8786845	0.910996414
Bosutinib	1.1090965	0.7804824	1.0985411	0.9776483	0.910118767
Imiquimod	1.0991822	0.8491946	1.2026013	0.9917523	0.887904663
Abiraterone	1.0443955	0.846116	1.1087497	1.1029108	0.854792766
