# Supplementary material for: Targeting the ubiquitin‐proteasome system in a pancreatic cancer subtype with hyperactive MYC
Source: Mol Oncol. 2020 Nov 8;14(12):3048–64. doi: 10.1002/1878-0261.12835 (PMC7718946; doi:10.1002/1878-0261.12835)
Supplement: Supplementary file 5 — Table S3. Pathways associated with MYC in common MYChigh PDAC. GSEA of pathways enriched in common MYChigh PDAC of the ICGC and the TCGA dataset. [file MOL2-14-3048-s005.pdf]

| Signature | ICGC | NES | NOM p-val | FDR q-val | TCGA | NES | NOM p-val | FDR q-val |
|---|---|---|---|---|---|---|---|---|
| GO_2_IRON_2_SULFUR_CLUSTER_BINDING | | 1.5546366 | 0.04752475 | 0.12653017 | | 1.8305562 | 0.00996016 | 0.03105208 |
| GO_4_IRON_4_SULFUR_CLUSTER_BINDING | | 1.8781075 | 0.00431035 | 0.02116238 | | 1.8316503 | 0.01483051 | 0.03103323 |
| GO_90S_PRERIBOSOME | | 1.8662984 | 0.00609756 | 0.02314922 | | 1.8861896 | 0.0078125 | 0.02100078 |
| GO_AEROBIC_ELECTRON_TRANSPORT_CHAIN | | 1.6586405 | 0.02335456 | 0.07452937 | | 1.7459227 | 0.00217391 | 0.05520465 |
| GO_AEROBIC_RESPIRATION | | 1.9587013 | 0.01890756 | 0.01483259 | | 1.9701035 | 0.0063425 | 0.01201548 |
| GO_AMINO_ACID_ACTIVATION | | 2.0083132 | 0.00424629 | 0.01287796 | | 2.0055063 | 0.00972763 | 0.00990288 |
| GO_ANAPHASE_PROMOTING_COMPLEX | | 1.7914375 | 0.00212314 | 0.03398622 | | 1.8604335 | 0.00606061 | 0.02421543 |
| GO_ANAPHASE_PROMOTING_COMPLEX_DEPENDENT_CATABOLIC_PROCESS | | 1.8996791 | 0 | 0.01870802 | | 2.073564 | 0 | 0.00683658 |
| GO_ANTIGEN_PROCESSING_AND_PRESENTATION_OF_EXOGENOUS_PEPTIDE_ANTIGEN_VIA_MHC_CLASS_I | | 1.5702072 | 0.09255533 | 0.11812779 | | 1.8086798 | 0.01807229 | 0.03681691 |
| GO_ANTIGEN_PROCESSING_AND_PRESENTATION_OF_PEPTIDE_ANTIGEN_VIA_MHC_CLASS_I | | 1.5075994 | 0.12525253 | 0.1587431 | | 1.5050008 | 0.10176125 | 0.19435759 |
| GO_APOPTOTIC_MITOCHONDRIAL_CHANGES | | 1.6247365 | 0.01310044 | 0.08969446 | | 1.5322015 | 0.01688555 | 0.17342627 |
| GO_ATP_SYNTHESIS_COUPLED_ELECTRON_TRANSPORT | | 1.9090444 | 0.00847458 | 0.01781047 | | 1.978608 | 0 | 0.01173385 |
| GO_BASE_EXCISION_REPAIR | | 1.7991501 | 0.00662252 | 0.03281886 | | 1.6633313 | 0.03571429 | 0.09130037 |
| GO_CAJAL_BODY | | 1.7672838 | 0.00672646 | 0.03885208 | | 1.4467956 | 0.0985577 | 0.24274197 |
| GO_CATALYTIC_ACTIVITY_ACTING_ON_A_RRNA | | 1.7463319 | 0.01276596 | 0.04473388 | | 2.0724566 | 0.00200401 | 0.00665854 |
| GO_CATALYTIC_ACTIVITY_ACTING_ON_DNA | | 1.9750601 | 0.00428266 | 0.01349506 | | 1.4532487 | 0.1124031 | 0.23920481 |
| GO_CATALYTIC_ACTIVITY_ACTING_ON_RNA | | 2.0996804 | 0.00422833 | 0.00722887 | | 1.9677322 | 0.00403226 | 0.01177533 |
| GO_CATALYTIC_STEP_2_SPLICEOSOME | | 2.189515 | 0.00215517 | 0.00373597 | | 1.8721489 | 0.02231237 | 0.02278461 |
| GO_CELL_CYCLE_DNA_REPLICATION | | 1.8440794 | 0.00858369 | 0.02634763 | | 1.6371154 | 0.07524753 | 0.10547344 |
| GO_CELL_CYCLE_G2_M_PHASE_TRANSITION | | 1.9311495 | 0.00662252 | 0.01691992 | | 1.6866989 | 0.03118908 | 0.08127189 |
| GO_CELL_REDOX_HOMEOSTASIS | | 1.4075692 | 0.12103175 | 0.24583554 | | 1.4440414 | 0.07392197 | 0.24563305 |
| GO_CELLULAR_COMPONENT_DISASSEMBLY | | 1.6931647 | 0.00617284 | 0.06115709 | | 1.8823427 | 0 | 0.0215491 |
| GO_CELLULAR_METABOLIC_COMPOUND_SALVAGE | | 1.5438282 | 0.02964427 | 0.1331458 | | 1.5519315 | 0.04545455 | 0.15834711 |
| GO_CELLULAR_PROTEIN_COMPLEX_DISASSEMBLY | | 1.9894688 | 0.00213675 | 0.01319396 | | 2.1009376 | 0 | 0.00637527 |
| GO_CELLULAR_RESPIRATION | | 1.9116265 | 0.01301519 | 0.01747709 | | 2.1236522 | 0 | 0.0055194 |
| GO_CELLULAR_RESPONSE_TO_OXYGEN_LEVELS | | 1.5694808 | 0.04661017 | 0.11812197 | | 1.5916075 | 0.00785855 | 0.13017248 |
| GO_CHAPERONE_BINDING | | 1.4569552 | 0.06029106 | 0.20116156 | | 1.5941439 | 0.03258656 | 0.12908132 |
| GO_CHAPERONE_COMPLEX | | 1.8484074 | 0.0059761 | 0.02586903 | | 1.7668012 | 0.02070393 | 0.04820181 |
| GO_CHAPERONE_MEDIATED_PROTEIN_FOLDING | | 1.646454 | 0.0349076 | 0.07907362 | | 1.4855691 | 0.08403362 | 0.20991012 |
| GO_CHROMOSOME_SEPARATION | | 1.8884473 | 0.00643777 | 0.01995567 | | 1.4835222 | 0.1326923 | 0.21085972 |
| GO_CIS_TRANS_ISOMERASE_ACTIVITY | | 1.6616777 | 0.02531646 | 0.07315822 | | 1.9003131 | 0 | 0.01837062 |
| GO_CLEAVAGE_INVOLVED_IN_RRNA_PROCESSING | | 1.927207 | 0 | 0.01706572 | | 1.9462715 | 0.00421053 | 0.01339045 |
| GO_CONDENSED_CHROMOSOME_CENTROMERIC_REGION | | 1.908657 | 0.00212766 | 0.0177083 | | 1.4861537 | 0.12871288 | 0.2097176 |
| GO_COTRANSLATIONAL_PROTEIN_TARGETING_TO_MEMBRANE | | 1.8328004 | 0.01372549 | 0.02758209 | | 1.9549553 | 0 | 0.01268143 |
| GO_CYTOCHROME_COMPLEX | | 1.6537461 | 0.04408818 | 0.07639154 | | 1.8530359 | 0.00443459 | 0.02582339 |
| GO_CYTOCHROME_COMPLEX_ASSEMBLY | | 1.8497947 | 0.00214133 | 0.02585637 | | 1.9598349 | 0 | 0.01229182 |
| GO_CYTOPLASMIC_TRANSLATION | | 2.0662992 | 0.0060241 | 0.00829011 | | 2.036124 | 0.00609756 | 0.00788216 |
| GO_CYTOPLASMIC_TRANSLATIONAL_INITIATION | | 1.9730803 | 0 | 0.01357926 | | 1.960311 | 0.00607288 | 0.01243657 |
| GO_CYTOSOLIC_LARGE_RIBOSOMAL_SUBUNIT | | 1.7885567 | 0.00389864 | 0.03444242 | | 2.0249257 | 0 | 0.00840269 |
| GO_CYTOSOLIC_PART | | 2.1912854 | 0.00198807 | 0.00374339 | | 2.2391512 | 0 | 0.00842333 |
| GO_CYTOSOLIC_RIBOSOME | | 1.8334333 | 0.01934236 | 0.02763226 | | 2.0519898 | 0 | 0.00760596 |
| GO_CYTOSOLIC_SMALL_RIBOSOMAL_SUBUNIT | | 1.7661823 | 0.01740812 | 0.03899746 | | 1.920439 | 0.00652174 | 0.01574955 |
| GO_DAMAGED_DNA_BINDING | | 1.8893907 | 0.00898876 | 0.01996675 | | 1.4742802 | 0.078125 | 0.21919294 |
| GO_DEOXYRIBONUCLEASE_ACTIVITY | | 1.7681144 | 0.01094092 | 0.03875539 | | 1.5850382 | 0.05168986 | 0.13479969 |
| GO_DEOXYRIBOSE_PHOSPHATE_CATABOLIC_PROCESS | | 1.5874077 | 0.03821656 | 0.10859895 | | 1.8053352 | 0.0040568 | 0.03754176 |
| GO_DISULFIDE_OXIDOREDUCTASE_ACTIVITY | | 1.6864059 | 0.02970297 | 0.06334098 | | 1.924583 | 0 | 0.01540211 |
| GO_DNA_BIOSYNTHETIC_PROCESS | | 1.9285553 | 0.00218818 | 0.01710591 | | 1.6534481 | 0.0239521 | 0.09628551 |
| GO_DNA_DAMAGE_RESPONSE_DETECTION_OF_DNA_DAMAGE | | 1.8489181 | 0.00847458 | 0.02583457 | | 1.9040192 | 0.00200401 | 0.01785688 |
| GO_DNA_DEPENDENT_DNA_REPLICATION | | 1.923255 | 0.01284797 | 0.01694357 | | 1.5653175 | 0.09823183 | 0.14854604 |
| GO_DNA_HELICASE_ACTIVITY | | 1.7863003 | 0.01758242 | 0.03498582 | | 1.6330994 | 0.02244898 | 0.10744654 |
| GO_DNA_POLYMERASE_BINDING | | 1.7268913 | 0.01452282 | 0.05042128 | | 1.7880663 | 0.01443299 | 0.04230921 |
| GO_DNA_REPLICATION_INITIATION | | 1.767758 | 0.01515152 | 0.0387636 | | 1.7515402 | 0.02208835 | 0.05322875 |
| GO_DNA_STRAND_ELONGATION | | 1.5571893 | 0.08351178 | 0.12483447 | | 1.4596456 | 0.12883435 | 0.23385483 |
| GO_DNA_TEMPLATED_TRANSCRIPTION_ELONGATION | | 2.2400477 | 0.00210971 | 0.00361458 | | 1.5418048 | 0.0625 | 0.16607438 |
| GO_DNA_TEMPLATED_TRANSCRIPTION_TERMINATION | | 2.1531723 | 0.00215054 | 0.00449661 | | 1.7928369 | 0.0139165 | 0.04083401 |
| GO_ELECTRON_TRANSPORT_CHAIN | | 1.5733513 | 0.06722689 | 0.11655197 | | 2.1021428 | 0 | 0.00640809 |
| GO_ENDODEOXYRIBONUCLEASE_ACTIVITY | | 1.7145984 | 0.01511879 | 0.05419442 | | 1.625722 | 0.02868852 | 0.11012238 |
| GO_ENDONUCLEASE_ACTIVITY | | 1.5651922 | 0.0465587 | 0.12057386 | | 1.8746098 | 0 | 0.02266256 |
| GO_ENDONUCLEASE_ACTIVITY_ACTIVE_WITH_EITHER_RIBO_OR_DEOXYRIBONUCLEIC_ACIDS_AND_PRODUCING_5_PHOSPHOMONOESTERS | | 1.933555 | 0.00420168 | 0.0167222 | | 2.0874212 | 0 | 0.00713616 |
| GO_ENDONUCLEASE_COMPLEX | | 1.9802613 | 0 | 0.01309578 | | 1.781354 | 0.00606061 | 0.04404187 |
| GO_ENDOPEPTIDASE_COMPLEX | | 1.9471263 | 0.00206186 | 0.01571803 | | 2.0092664 | 0 | 0.00984862 |
| GO_ENDORIBONUCLEASE_ACTIVITY | | 1.5107265 | 0.05636743 | 0.15654269 | | 1.9835452 | 0.00210526 | 0.01167787 |
| GO_ENDORIBONUCLEASE_ACTIVITY_PRODUCING_5_PHOSPHOMONOESTERS | | 1.922281 | 0.00204499 | 0.01681793 | | 1.9998231 | 0.00208768 | 0.01034526 |
| GO_ENERGY_DERIVATION_BY_OXIDATION_OF_ORGANIC_COMPOUNDS | | 1.7602427 | 0.01923077 | 0.04085524 | | 2.0379908 | 0 | 0.00780972 |
| GO_ERROR_FREE_TRANSLESION_SYNTHESIS | | 1.7249316 | 0.00833333 | 0.05099789 | | 1.6703199 | 0.03171247 | 0.08851618 |
| GO_ERROR_PRONE_TRANSLESION_SYNTHESIS | | 1.8029855 | 0.01046025 | 0.03218533 | | 1.5298263 | 0.082 | 0.17546372 |
| GO_ESTABLISHMENT_OF_PROTEIN_LOCALIZATION_TO_CHROMOSOME | | 1.995776 | 0.00204918 | 0.01304769 | | 1.738035 | 0.0221328 | 0.05837833 |
| GO_ESTABLISHMENT_OF_PROTEIN_LOCALIZATION_TO_ENDOPLASMIC_RETICULUM | | 1.937211 | 0.01008065 | 0.01634823 | | 2.0085297 | 0 | 0.00982766 |
| GO_ESTABLISHMENT_OF_PROTEIN_LOCALIZATION_TO_MEMBRANE | | 1.8069315 | 0.008 | 0.03155933 | | 2.217026 | 0 | 0.00652453 |
| GO_ESTABLISHMENT_OF_PROTEIN_LOCALIZATION_TO_MITOCHONDRIAL_MEMBRANE | | 1.8276556 | 0.00206612 | 0.02812632 | | 1.880299 | 0 | 0.02190515 |
| GO_ESTABLISHMENT_OF_PROTEIN_LOCALIZATION_TO_TELOMERE | | 1.8641248 | 0.00206186 | 0.02346929 | | 1.6697475 | 0.03992016 | 0.08862737 |
| GO_EUKARYOTIC_48S_PREINITIATION_COMPLEX | | 1.7928109 | 0 | 0.0336195 | | 1.7936659 | 0.00798403 | 0.04069156 |
| GO_EUKARYOTIC_TRANSLATION_INITIATION_FACTOR_3_COMPLEX | | 1.8314188 | 0 | 0.02768957 | | 1.7133452 | 0.02217742 | 0.06961399 |
| GO_EXODEOXYRIBONUCLEASE_ACTIVITY | | 1.6334605 | 0.0372807 | 0.08532978 | | 1.620989 | 0.03875969 | 0.11284037 |
| GO_EXON_EXON_JUNCTION_COMPLEX | | 1.7207321 | 0.02226721 | 0.05240382 | | 1.4371762 | 0.1 | 0.25015232 |
| GO_EXOSOME_RNASE_COMPLEX_ | | 1.9538167 | 0.0021645 | 0.01520247 | | 1.8516189 | 0.00201613 | 0.0260626 |
| GO_FEMALE_MEIOTIC_NUCLEAR_DIVISION | | 1.592025 | 0.07157895 | 0.10645982 | | 1.5479482 | 0.08964144 | 0.16016409 |
| GO_FICOLIN_1_RICH_GRANULE | | 1.693058 | 0.04781705 | 0.0611 | | 1.751067 | 0.01330799 | 0.05323966 |
| GO_GENERATION_OF_PRECURSOR_METABOLITES_AND_ENERGY | | 1.553867 | 0.04338843 | 0.12677354 | | 1.9426553 | 0 | 0.01370454 |
| GO_GLOBAL_GENOME_NUCLEOTIDE_EXCISION_REPAIR | | 1.950357 | 0.00860215 | 0.01544956 | | 1.519522 | 0.05443548 | 0.18399344 |
| GO_GLYCOSYL_COMPOUND_BIOSYNTHETIC_PROCESS | | 1.6646495 | 0.02564103 | 0.07199428 | | 1.8963711 | 0.00401606 | 0.01902106 |
| GO_HEMATOPOIETIC_STEM_CELL_DIFFERENTIATION | | 1.6960298 | 0.03488372 | 0.05990372 | | 1.8795451 | 0.00787402 | 0.02179604 |
| GO_HISTONE_EXCHANGE | | 1.7735453 | 0.01742919 | 0.03780221 | | 1.4818102 | 0.11471862 | 0.21253145 |
| GO_INNER_MITOCHONDRIAL_MEMBRANE_ORGANIZATION | | 1.8540591 | 0.01101322 | 0.02516892 | | 1.9737797 | 0.00205761 | 0.01184356 |
| GO_INNER_MITOCHONDRIAL_MEMBRANE_PROTEIN_COMPLEX | | 1.9916044 | 0.00426439 | 0.01312152 | | 2.0244234 | 0 | 0.00833269 |
| GO_INTERLEUKIN_1_MEDIATED_SIGNALING_PATHWAY | | 2.0920637 | 0 | 0.00722003 | | 1.6882825 | 0.02713178 | 0.08056341 |
| GO_INTERSTRAND_CROSS_LINK_REPAIR | | 1.9147025 | 0.00220264 | 0.0174138 | | 1.4422898 | 0.12138728 | 0.24625412 |
| GO_INTRACELLULAR_PROTEIN_TRANSMEMBRANE_TRANSPORT | | 2.000829 | 0 | 0.01264928 | | 1.9713533 | 0.00200401 | 0.01208168 |
| GO_INTRAMOLECULAR_TRANSFERASE_ACTIVITY | | 1.7942084 | 0.01079914 | 0.03348633 | | 1.5252091 | 0.05544554 | 0.17917624 |
| GO_INTRINSIC_COMPONENT_OF_MITOCHONDRIAL_INNER_MEMBRANE | | 1.7368808 | 0.01118568 | 0.04738492 | | 2.0655956 | 0.00197629 | 0.00707175 |
| GO_INTRINSIC_COMPONENT_OF_MITOCHONDRIAL_MEMBRANE | | 1.6103014 | 0.05010893 | 0.09671661 | | 1.9228024 | 0.00591716 | 0.01562059 |
| GO_IRON_SULFUR_CLUSTER_ASSEMBLY | | 1.8070924 | 0.00668151 | 0.03159579 | | 1.9043012 | 0 | 0.01792404 |
| GO_ISOMERASE_ACTIVITY | | 1.5751219 | 0.02136752 | 0.11558973 | | 1.835956 | 0 | 0.03031624 |
| GO_LARGE_RIBOSOMAL_SUBUNIT | | 2.0034301 | 0.00203252 | 0.01286257 | | 2.083209 | 0 | 0.00694732 |
| GO_LIGASE_ACTIVITY_FORMING_CARBON_OXYGEN_BONDS | | 1.9029763 | 0.0043573 | 0.01838177 | | 1.9853064 | 0.00596422 | 0.01154037 |
| GO_MATURATION_OF_5_8S_RRNA | | 1.9259416 | 0 | 0.01706409 | | 1.9834164 | 0.00208333 | 0.01159413 |
| GO_MATURATION_OF_5_8S_RRNA_FROM_TRICISTRONIC_RRNA_TRANSCRIPT_SSU_RRNA_5_8S_RRNA_LSU_RRNA_ | | 1.9346548 | 0 | 0.01674035 | | 1.9684064 | 0.00419287 | 0.01203176 |
| GO_MATURATION_OF_LSU_RRNA | | 1.9281245 | 0 | 0.01706805 | | 1.939521 | 0 | 0.01389173 |
| GO_MATURATION_OF_SSU_RRNA | | 2.0549889 | 0 | 0.00900236 | | 2.0286446 | 0.002 | 0.00830753 |
| GO_MATURATION_OF_SSU_RRNA_FROM_TRICISTRONIC_RRNA_TRANSCRIPT_SSU_RRNA_5_8S_RRNA_LSU_RRNA_ | | 1.9844301 | 0 | 0.01302605 | | 2.0045562 | 0 | 0.00991967 |
| GO_METAL_CLUSTER_BINDING | | 1.7941837 | 0.00631579 | 0.03341325 | | 2.0299377 | 0 | 0.00826139 |
| GO_METAPHASE_ANAPHASE_TRANSITION_OF_CELL_CYCLE | | 1.7797961 | 0.02132196 | 0.03628285 | | 1.4842916 | 0.1362764 | 0.21093006 |
| GO_METAPHASE_PLATE_CONGRESSION | | 1.847305 | 0.00652174 | 0.02606935 | | 1.5689954 | 0.07170542 | 0.14593206 |
| GO_MISMATCH_REPAIR | | 1.4469527 | 0.08478261 | 0.20902188 | | 1.4594288 | 0.08510638 | 0.23356476 |
| GO_MITOCHONDRIAL_ELECTRON_TRANSPORT_NADH_TO_UBIQUINONE | | 1.6293607 | 0.06622516 | 0.08720037 | | 1.9308388 | 0 | 0.01510363 |
| GO_MITOCHONDRIAL_GENE_EXPRESSION | | 2.0579221 | 0 | 0.00903079 | | 2.1319304 | 0 | 0.00570553 |
| GO_MITOCHONDRIAL_MATRIX | | 1.8472384 | 0.01082251 | 0.02599322 | | 2.2181787 | 0 | 0.00765207 |
| GO_MITOCHONDRIAL_MEMBRANE_ORGANIZATION | | 1.9874864 | 0.00221239 | 0.0131214 | | 1.8024865 | 0.00191571 | 0.03784418 |
| GO_MITOCHONDRIAL_MEMBRANE_PART | | 1.8342129 | 0.0131579 | 0.02770652 | | 2.1366553 | 0 | 0.00636864 |
| GO_MITOCHONDRIAL_PROTEIN_COMPLEX | | 2.087385 | 0 | 0.00753432 | | 2.1047716 | 0 | 0.00625337 |
| GO_MITOCHONDRIAL_RESPIRATORY_CHAIN_COMPLEX_ASSEMBLY | | 1.9253925 | 0.00444445 | 0.01687264 | | 2.0453324 | 0 | 0.00749442 |
| GO_MITOCHONDRIAL_RESPIRATORY_CHAIN_COMPLEX_I | | 1.6261784 | 0.05777778 | 0.08884403 | | 1.9666957 | 0 | 0.01179943 |
| GO_MITOCHONDRIAL_RNA_METABOLIC_PROCESS | | 1.8401432 | 0.02542373 | 0.02691623 | | 1.7976534 | 0.01247401 | 0.03937479 |
| GO_MITOCHONDRIAL_TRANSLATION | | 2.04209 | 0 | 0.01072653 | | 2.126935 | 0 | 0.00538819 |
| GO_MITOCHONDRIAL_TRANSLATIONAL_TERMINATION | | 2.0374904 | 0 | 0.01082413 | | 2.050288 | 0 | 0.00749586 |
| GO_MITOCHONDRIAL_TRANSMEMBRANE_TRANSPORT | | 1.6178974 | 0.05010893 | 0.09333035 | | 1.808093 | 0.00645161 | 0.03685258 |
| GO_MITOCHONDRIAL_TRANSPORT | | 1.8196676 | 0.01505376 | 0.02937791 | | 1.897619 | 0.00198807 | 0.01890703 |
| GO_MITOCHONDRION_ORGANIZATION | | 1.9861525 | 0 | 0.01302283 | | 2.077669 | 0 | 0.00680881 |
| GO_MITOTIC_METAPHASE_PLATE_CONGRESSION | | 1.8108593 | 0.00428266 | 0.03091179 | | 1.7309953 | 0.02879079 | 0.06158856 |
| GO_MITOTIC_NUCLEAR_DIVISION | | 1.9315755 | 0.00431965 | 0.01692344 | | 1.4609115 | 0.11218569 | 0.23337938 |
| GO_MITOTIC_SISTER_CHROMATID_SEGREGATION | | 1.9033178 | 0.00643777 | 0.01838307 | | 1.4971949 | 0.11264822 | 0.20142198 |
| GO_MLL1_2_COMPLEX | | 1.8356984 | 0.01902749 | 0.02740547 | | 1.5773841 | 0.04743083 | 0.14053869 |
| GO_MRNA_5_UTR_BINDING | | 2.1279755 | 0 | 0.00623267 | | 1.5394182 | 0.02542373 | 0.16808707 |
| GO_MRNA_CIS_SPLICING_VIA_SPLICEOSOME | | 1.5059924 | 0.11688311 | 0.15962625 | | 1.6982656 | 0.02631579 | 0.07553295 |
| GO_NADH_DEHYDROGENASE_ACTIVITY | | 1.7113658 | 0.02407002 | 0.05489301 | | 1.4532506 | 0.09839817 | 0.23976888 |
| GO_NADH_DEHYDROGENASE_COMPLEX_ASSEMBLY | | 1.8692024 | 0.00867679 | 0.02269838 | | 1.9296266 | 0 | 0.01510238 |
| GO_NCRNA_3_END_PROCESSING | | 2.0049105 | 0.00212766 | 0.01298959 | | 2.138455 | 0 | 0.00672035 |
| GO_NCRNA_METABOLIC_PROCESS | | 2.1965227 | 0.00215983 | 0.00371978 | | 1.6307688 | 0.03658536 | 0.10813277 |
| GO_NCRNA_PROCESSING | | 2.2285333 | 0.0021645 | 0.00298222 | | 2.24441 | 0.00208333 | 0.01187643 |
| GO_NCRNA_TRANSCRIPTION | | 2.2272177 | 0.00205339 | 0.00286752 | | 1.6797867 | 0.01698113 | 0.08426998 |
| GO_NEGATIVE_REGULATION_OF_CELL_CYCLE_G2_M_PHASE_TRANSITION | | 1.9621655 | 0 | 0.01464971 | | 1.9632004 | 0.00394477 | 0.01215496 |
| GO_NEGATIVE_REGULATION_OF_CELL_CYCLE_PHASE_TRANSITION | | 2.009199 | 0 | 0.01294595 | | 1.6806107 | 0.02895753 | 0.08393965 |
| GO_NEGATIVE_REGULATION_OF_CELL_CYCLE_PROCESS | | 2.0046508 | 0 | 0.01290752 | | 1.5593582 | 0.05447471 | 0.15316132 |
| GO_NEGATIVE_REGULATION_OF_MITOTIC_CELL_CYCLE | | 2.010031 | 0 | 0.0130065 | | 1.58516 | 0.05113636 | 0.13509119 |
| GO_NEGATIVE_REGULATION_OF_RNA_SPLICING | | 1.6088134 | 0.06095238 | 0.09713763 | | 1.4357954 | 0.11410788 | 0.24874553 |
| GO_NEGATIVE_REGULATION_OF_UBIQUITIN_PROTEIN_TRANSFERASE_ACTIVITY | | 1.6892977 | 0.02731093 | 0.06192495 | | 1.5725653 | 0.05544148 | 0.14333168 |

| | | | | | | |
|---|---|---|---|---|---|---|
| GO_NF_KAPPAB_BINDING | 1.9233649 | 0 | 0.01697921 | 1.6410003 | 0.02816901 | 0.10320844 |
| GO_NUCLEAR_DNA_REPLICATION | 1.8187983 | 0.01086957 | 0.02952212 | 1.7856219 | 0.0259481 | 0.04285713 |
| GO_NUCLEAR_ENVELOPE_ORGANIZATION | 1.7314293 | 0.03501094 | 0.04904145 | 1.4495665 | 0.07899807 | 0.2407751 |
| GO_NUCLEAR_ENVELOPE_REASSEMBLY | 1.6519934 | 0.0237069 | 0.0767554 | 1.6137468 | 0.03522505 | 0.11658422 |
| GO_NUCLEAR_EXOSOME_RNASE_COMPLEX_ | 1.7324094 | 0.02335456 | 0.04883638 | 1.9292631 | 0 | 0.01507779 |
| GO_NUCLEAR_EXPORT | 2.3690681 | 0 | 0.00278036 | 1.5103556 | 0.0724346 | 0.19023578 |
| GO_NUCLEAR_TRANSCRIBED_MRNA_CATABOLIC_PROCESS | 2.2456336 | 0.00208768 | 0.0034168 | 2.0846853 | 0.00829876 | 0.00697786 |
| GO_NUCLEAR_TRANSCRIBED_MRNA_CATABOLIC_PROCESS_DEADENYLATION_DEPENDENT_DECAY | 1.7600857 | 0.02966102 | 0.04080775 | 1.560953 | 0.03960396 | 0.15201078 |
| GO_NUCLEAR_TRANSCRIBED_MRNA_CATABOLIC_PROCESS_EXONUCLEOLYTIC | 1.9012892 | 0.00840336 | 0.01865215 | 1.9272076 | 0.00996016 | 0.01514675 |
| GO_NUCLEAR_TRANSCRIBED_MRNA_CATABOLIC_PROCESS_NONSENSE_MEDIATED_DECAY | 2.0451658 | 0.00200803 | 0.01040872 | 2.0449262 | 0.00211417 | 0.00743299 |
| GO_NUCLEAR_UBIQUITIN_LIGASE_COMPLEX | 1.8159461 | 0.01324503 | 0.02993145 | 1.509472 | 0.04305284 | 0.19018213 |
| GO_NUCLEASE_ACTIVITY | 1.6996152 | 0.01914894 | 0.05880825 | 1.6271704 | 0.01239669 | 0.11042336 |
| GO_NUCLEIC_ACID_PHOSPHODIESTER_BOND_HYDROLYSIS | 1.872179 | 0.00867679 | 0.02212491 | 1.7712934 | 0.004 | 0.04770253 |
| GO_NUCLEOBASE_BIOSYNTHETIC_PROCESS | 1.7310221 | 0.01769912 | 0.04898916 | 1.6783388 | 0.03958333 | 0.08492755 |
| GO_NUCLEOBASE_CONTAINING_SMALL_MOLECULE_CATABOLIC_PROCESS | 1.4668792 | 0.03869654 | 0.19297774 | 1.8839909 | 0 | 0.02136374 |
| GO_NUCLEOID | 2.0140438 | 0 | 0.01261005 | 1.9678727 | 0.00616016 | 0.01185984 |
| GO_NUCLEOLAR_PART | 1.8013424 | 0.01077586 | 0.03253086 | 1.8340183 | 0.0077821 | 0.03075712 |
| GO_NUCLEOSIDE_MONOPHOSPHATE_BIOSYNTHETIC_PROCESS | 1.8203661 | 0.00649351 | 0.02935173 | 2.001296 | 0 | 0.01028919 |
| GO_NUCLEOSIDE_SALVAGE | 1.6428069 | 0.0101626 | 0.08083905 | 1.4873295 | 0.07905138 | 0.20874988 |
| GO_NUCLEOSIDE_TRIPHOSPHATE_BIOSYNTHETIC_PROCESS | 1.667621 | 0.02105263 | 0.07078502 | 1.8789903 | 0.0019685 | 0.02166957 |
| GO_NUCLEOTIDE_EXCISION_REPAIR | 2.183258 | 0 | 0.0036901 | 1.9215455 | 0.00595238 | 0.01576211 |
| GO_NUCLEOTIDE_EXCISION_REPAIR_DNA_DAMAGE_RECOGNITION | 1.7783326 | 0.01054852 | 0.03649476 | 1.8313439 | 0.01489362 | 0.03094923 |
| GO_NUCLEOTIDE_EXCISION_REPAIR_DNA_DUPLEX_UNWINDING | 1.826784 | 0.00840336 | 0.02815636 | 1.7021626 | 0.01616162 | 0.07411315 |
| GO_NUCLEOTIDE_EXCISION_REPAIR_DNA_GAP_FILLING | 1.6574824 | 0.04458599 | 0.07512108 | 1.6715283 | 0.03861789 | 0.08804886 |
| GO_NUCLEOTIDE_EXCISION_REPAIR_DNA_INCISION | 2.0065653 | 0.00218818 | 0.01311337 | 2.0611687 | 0.0019305 | 0.00717928 |
| GO_NUCLEOTIDE_EXCISION_REPAIR_PREINCISION_COMPLEX_ASSEMBLY | 1.8891623 | 0.00646552 | 0.01994404 | 1.8210578 | 0.004 | 0.03332983 |
| GO_NUCLEOTIDE_EXCISION_REPAIR_PREINCISION_COMPLEX_STABILIZATION | 1.8392164 | 0.01072961 | 0.0270413 | 1.7655567 | 0.01619433 | 0.04848409 |
| GO_NUCLEOTIDYLTRANSFERASE_ACTIVITY | 2.0265138 | 0.0022173 | 0.01151852 | 1.8330625 | 0.00584795 | 0.03072586 |
| GO_O_METHYLTRANSFERASE_ACTIVITY | 1.439917 | 0.11316872 | 0.21418722 | 1.6729343 | 0.01840491 | 0.08740791 |
| GO_OLIGOSACCHARIDE_LIPID_INTERMEDIATE_BIOSYNTHETIC_PROCESS | 1.6417854 | 0.03586498 | 0.0810796 | 1.4360046 | 0.11881188 | 0.24899878 |
| GO_ORGANELLAR_LARGE_RIBOSOMAL_SUBUNIT | 1.9268824 | 0 | 0.01705411 | 1.9576352 | 0 | 0.01251926 |
| GO_ORGANELLAR_RIBOSOME | 2.032424 | 0 | 0.01103879 | 2.005667 | 0 | 0.00999968 |
| GO_ORGANELLAR_SMALL_RIBOSOMAL_SUBUNIT | 1.9890507 | 0 | 0.01316798 | 1.977039 | 0 | 0.01177381 |
| GO_ORGANELLE_ENVELOPE_LUMEN | 1.9409759 | 0.00409836 | 0.01633011 | 2.09498 | 0 | 0.00665314 |
| GO_ORGANELLE_INNER_MEMBRANE | 1.9245344 | 0.01084599 | 0.01701663 | 2.190692 | 0 | 0.00575882 |
| GO_OUTER_MITOCHONDRIAL_MEMBRANE_PROTEIN_COMPLEX | 1.9772155 | 0 | 0.01336573 | 1.7676538 | 0.00606061 | 0.04798727 |
| GO_OXIDATIVE_PHOSPHORYLATION | 1.8496555 | 0.01724138 | 0.02579306 | 2.0808995 | 0 | 0.0068305 |
| GO_OXIDOREDUCTASE_ACTIVITY_ACTING_ON_A_HEME_GROUP_OF_DONORS | 1.4377309 | 0.11632653 | 0.21513659 | 1.9106821 | 0.00224215 | 0.01707003 |
| GO_OXIDOREDUCTASE_ACTIVITY_ACTING_ON_NAD_P_H_QUINONE_OR_SIMILAR_COMPOUND_AS_ACCEPTOR | 1.5014703 | 0.08333334 | 0.16338961 | 1.9791368 | 0 | 0.01186213 |
| GO_OXIDOREDUCTASE_COMPLEX | 1.5858555 | 0.07484408 | 0.10967447 | 2.208679 | 0 | 0.00597146 |
| GO_PEPTIDASE_COMPLEX | 2.0269334 | 0 | 0.01153572 | 2.1411657 | 0 | 0.00723096 |
| GO_PEPTIDYL_ARGININE_MODIFICATION | 1.4900883 | 0.06099815 | 0.1729746 | 1.477367 | 0.0389016 | 0.21646981 |
| GO_PEPTIDYL_PROLINE_MODIFICATION | 1.6059327 | 0.05708245 | 0.09882093 | 1.7507063 | 0.0102459 | 0.05313384 |
| GO_PIGMENT_BIOSYNTHETIC_PROCESS | 1.719225 | 0.01670146 | 0.05284357 | 1.5134153 | 0.02946955 | 0.18926182 |
| GO_POLYSOMAL_RIBOSOME | 1.795513 | 0 | 0.03345429 | 1.8640648 | 0.00840336 | 0.02377011 |
| GO_POLYSOME | 2.0189724 | 0.00588235 | 0.01216475 | 2.0469518 | 0.00399202 | 0.00759622 |
| GO_POSITIVE_REGULATION_OF_CELLULAR_AMIDE_METABOLIC_PROCESS | 1.5918329 | 0.03448276 | 0.10640177 | 1.6489224 | 0.01375246 | 0.09823954 |
| GO_POSITIVE_REGULATION_OF_DNA_BIOSYNTHETIC_PROCESS | 1.5899321 | 0.04602511 | 0.10699172 | 1.54918 | 0.06370656 | 0.15983294 |
| GO_POSITIVE_REGULATION_OF_PROTEIN_LOCALIZATION_TO_NUCLEUS | 1.7649574 | 0.01709402 | 0.03936537 | 1.5480102 | 0.04069768 | 0.16054936 |
| GO_POSITIVE_REGULATION_OF_TELOMERASE_ACTIVITY | 1.4699312 | 0.08991228 | 0.19049431 | 1.5531958 | 0.07794677 | 0.15750101 |
| GO_POSITIVE_REGULATION_OF_TELOMERASE_RNA_LOCALIZATION_TO_CAJAL_BODY | 1.7185833 | 0.00817996 | 0.05300026 | 1.9769957 | 0.00192678 | 0.01166554 |
| GO_POSITIVE_REGULATION_OF_VIRAL_PROCESS | 1.8506109 | 0.01844262 | 0.0256935 | 1.4655552 | 0.03442029 | 0.22856499 |
| GO_POSITIVE_REGULATION_OF_VIRAL_TRANSCRIPTION | 2.193418 | 0.00200401 | 0.0038437 | 1.5761999 | 0.02761341 | 0.14074501 |
| GO_POSTREPLICATION_REPAIR | 1.969971 | 0.00434783 | 0.01379843 | 1.6523377 | 0.03359684 | 0.09675098 |
| GO_PRECATALYTIC_SPLICEOSOME | 1.9453475 | 0.00420168 | 0.01580541 | 1.9572984 | 0.00618557 | 0.01248009 |
| GO_PRERIBOSOME | 2.0015123 | 0.00202429 | 0.01279714 | 2.0317957 | 0.00609756 | 0.00808153 |
| GO_PRERIBOSOME_LARGE_SUBUNIT_PRECURSOR | 1.7060698 | 0.00204082 | 0.0568016 | 1.9810989 | 0.00204082 | 0.01174159 |
| GO_PROTEASOMAL_PROTEIN_CATABOLIC_PROCESS | 1.8643802 | 0.00627615 | 0.02348525 | 1.4917773 | 0.02214022 | 0.20499627 |
| GO_PROTEASOMAL_UBIQUITIN_INDEPENDENT_PROTEIN_CATABOLIC_PROCESS | 1.5585196 | 0.05702648 | 0.1246532 | 1.7691929 | 0.00852879 | 0.04797814 |
| GO_PROTEASOME_ACCESSORY_COMPLEX | 1.7334505 | 0.00204082 | 0.04844138 | 1.8622715 | 0.00592885 | 0.02399346 |
| GO_PROTEASOME_CORE_COMPLEX | 1.6689172 | 0.00631579 | 0.07052892 | 1.7390951 | 0.00220751 | 0.05835803 |
| GO_PROTEIN_CONTAINING_COMPLEX_DISASSEMBLY | 1.9466709 | 0 | 0.01566645 | 2.053913 | 0 | 0.00753631 |
| GO_PROTEIN_DISULFIDE_OXIDOREDUCTASE_ACTIVITY | 1.8954461 | 0.00394477 | 0.01916402 | 1.7910824 | 0.00813008 | 0.0414159 |
| GO_PROTEIN_DNA_COMPLEX_SUBUNIT_ORGANIZATION | 1.8827263 | 0.01508621 | 0.02073055 | 1.4365296 | 0.08583691 | 0.24935175 |
| GO_PROTEIN_FOLDING | 1.8376025 | 0.01242236 | 0.02712501 | 1.9816844 | 0.00209644 | 0.01174834 |
| GO_PROTEIN_IMPORT_INTO_MITOCHONDRIAL_MATRIX | 1.8602245 | 0 | 0.02398159 | 1.9012705 | 0 | 0.01827472 |
| GO_PROTEIN_INSERTION_INTO_MEMBRANE | 1.8301889 | 0.0040568 | 0.02779682 | 1.7790365 | 0 | 0.04460175 |
| GO_PROTEIN_INSERTION_INTO_MITOCHONDRIAL_MEMBRANE | 1.8315679 | 0.00632911 | 0.02773637 | 1.6321154 | 0.00760456 | 0.10781675 |
| GO_PROTEIN_K11_LINKED_UBIQUITINATION | 1.6496907 | 0.04752066 | 0.07754524 | 1.7525613 | 0.02091255 | 0.05298182 |
| GO_PROTEIN_LOCALIZATION_TO_ENDOPLASMIC_RETICULUM | 1.8916347 | 0.02235772 | 0.01975925 | 2.118036 | 0 | 0.00575471 |
| GO_PROTEIN_LOCALIZATION_TO_MITOCHONDRION | 1.9650809 | 0.00210084 | 0.01422835 | 1.9787599 | 0.00199203 | 0.01182534 |
| GO_PROTEIN_MODIFICATION_BY_SMALL_PROTEIN_REMOVAL | 2.0329878 | 0 | 0.01114413 | 1.4366407 | 0.06976745 | 0.24977139 |
| GO_PROTEIN_NEDDYLATION | 1.7249324 | 0.01386139 | 0.05110851 | 1.5770901 | 0.05744681 | 0.14037375 |
| GO_PROTEIN_PEPTIDYL_PROLYL_ISOMERIZATION | 1.701808 | 0.04166667 | 0.05798405 | 1.8210669 | 0.00211417 | 0.03349479 |
| GO_PROTEIN_QUALITY_CONTROL_FOR_MISFOLDED_OR_INCOMPLETELY_SYNTHESIZED_PROTEINS | 1.5914953 | 0.04545455 | 0.10650186 | 1.4553559 | 0.05432596 | 0.23826046 |
| GO_PROTEIN_TARGETING | 1.9273317 | 0.0041841 | 0.01713257 | 2.1287537 | 0.00207039 | 0.00544101 |
| GO_PROTEIN_TARGETING_TO_MEMBRANE | 1.9539355 | 0.00595238 | 0.01529285 | 2.2445607 | 0 | 0.02375287 |
| GO_PROTEIN_TARGETING_TO_MITOCHONDRION | 1.9666901 | 0.00622407 | 0.01418276 | 2.1381721 | 0 | 0.00645831 |
| GO_PROTEIN_TRANSMEMBRANE_IMPORT_INTO_INTRACELLULAR_ORGANELLE | 1.9711045 | 0 | 0.01373525 | 1.8791935 | 0.00413223 | 0.0217176 |
| GO_PROTEIN_TRANSMEMBRANE_TRANSPORT | 1.9748048 | 0.00205339 | 0.01340614 | 2.038398 | 0.0020284 | 0.00786634 |
| GO_PROTEIN_TRANSPORTER_ACTIVITY | 1.6587024 | 0.0256917 | 0.07462262 | 1.6020724 | 0.01757813 | 0.12287636 |
| GO_PROTON_TRANSPORTING_TWO_SECTOR_ATPASE_COMPLEX | 1.5926589 | 0.0421941 | 0.10629695 | 1.4413953 | 0.10040984 | 0.2469202 |
| GO_PSEUDOURIDINE_SYNTHESIS | 1.896903 | 0.00212766 | 0.01897282 | 1.7206028 | 0.01803607 | 0.06599229 |
| GO_PURINE_NUCLEOSIDE_BIOSYNTHETIC_PROCESS | 1.4276378 | 0.08092485 | 0.2254352 | 1.8923898 | 0.00197629 | 0.01974089 |
| GO_PURINE_NUCLEOSIDE_MONOPHOSPHATE_BIOSYNTHETIC_PROCESS | 1.8114953 | 0 | 0.03078106 | 1.8668073 | 0.00842105 | 0.02341268 |
| GO_PYRIMIDINE_CONTAINING_COMPOUND_BIOSYNTHETIC_PROCESS | 1.4802861 | 0.05781585 | 0.1813439 | 1.8666629 | 0.00594059 | 0.02333478 |
| GO_PYRIMIDINE_NUCLEOSIDE_MONOPHOSPHATE_BIOSYNTHETIC_PROCESS | 1.6107582 | 0.04250559 | 0.09690166 | 1.6619028 | 0.02574257 | 0.09201698 |
| GO_PYRIMIDINE_NUCLEOTIDE_BIOSYNTHETIC_PROCESS | 1.5574156 | 0.03303965 | 0.12483344 | 1.9283013 | 0.00196464 | 0.01514806 |
| GO_PYRIMIDINE_RIBONUCLEOTIDE_BIOSYNTHETIC_PROCESS | 1.4740709 | 0.07272727 | 0.1869442 | 1.7012109 | 0.02028398 | 0.07437295 |
| GO_REGULATION_OF_CELL_CYCLE_ARREST | 1.6915 | 0.01059322 | 0.06140494 | 1.4716754 | 0.058 | 0.22178091 |
| GO_REGULATION_OF_CELL_CYCLE_G2_M_PHASE_TRANSITION | 1.9057531 | 0.01121076 | 0.01801452 | 1.7594093 | 0.02178218 | 0.05101793 |
| GO_REGULATION_OF_CELL_CYCLE_PHASE_TRANSITION | 1.9901812 | 0.004329 | 0.01318346 | 1.5015099 | 0.06576403 | 0.19743066 |
| GO_REGULATION_OF_CELLULAR_AMIDE_METABOLIC_PROCESS | 1.8395616 | 0.0021645 | 0.02702067 | 2.1819146 | 0 | 0.00563571 |
| GO_REGULATION_OF_CHROMOSOME_SEPARATION | 1.7771627 | 0.02547771 | 0.03679544 | 1.5134051 | 0.11516315 | 0.18877289 |
| GO_REGULATION_OF_DNA_BIOSYNTHETIC_PROCESS | 1.6565903 | 0.01793722 | 0.07525609 | 1.4532033 | 0.07378641 | 0.23869795 |
| GO_REGULATION_OF_DNA_TEMPLATED_TRANSCRIPTION_IN_RESPONSE_TO_STRESS | 1.8229514 | 0.01666667 | 0.02880438 | 1.7559134 | 0.01383399 | 0.05214544 |
| GO_REGULATION_OF_ESTABLISHMENT_OF_PLANAR_POLARITY | 1.5236623 | 0.07113821 | 0.14769147 | 1.6029171 | 0.04882813 | 0.12325236 |
| GO_REGULATION_OF_HEMATOPOIETIC_PROGENITOR_CELL_DIFFERENTIATION | 1.9520475 | 0.00203252 | 0.01535581 | 1.7072817 | 0.02539063 | 0.07266405 |
| GO_REGULATION_OF_MITOCHONDRIAL_OUTER_MEMBRANE_PERMEABILIZATION_INVOLVED_IN_APOPTOTIC_SIGNALING_PATHWAY | 1.7578232 | 0.01716738 | 0.04127476 | | | |
| GO_REGULATION_OF_MITOCHONDRIAL_TRANSLATION | 1.9585555 | 0 | 0.01473789 | 1.4794713 | 0.04036697 | 0.2143359 |
| GO_REGULATION_OF_MRNA_CATABOLIC_PROCESS | 1.8051983 | 0.01483051 | 0.03184892 | 1.8796582 | 0.00206612 | 0.02189971 |
| GO_REGULATION_OF_RELEASE_OF_CYTOCHROME_C_FROM_MITOCHONDRIA | 1.4047052 | 0.04862579 | 0.24903302 | 1.6745099 | 0.01778656 | 0.08691016 |
| GO_REGULATION_OF_STEM_CELL_DIFFERENTIATION | 1.498783 | 0.10060363 | 0.16538422 | 1.4933344 | 0.05836576 | 0.20376335 |
| GO_REGULATION_OF_TELOMERASE_ACTIVITY | 1.5337137 | 0.05111111 | 0.14033787 | 1.4997196 | 0.07251909 | 0.1990469 |
| GO_REGULATION_OF_TRANSCRIPTION_FROM_RNA_POLYMERASE_II_PROMOTER_IN_RESPONSE_TO_HYPOXIA | 2.0926378 | 0 | 0.00733306 | 1.953071 | 0.00595238 | 0.01270493 |
| GO_REGULATION_OF_TRANSLATIONAL_FIDELITY | 1.6982397 | 0.03043478 | 0.05923051 | 1.8032478 | 0.01030928 | 0.0377416 |
| GO_REGULATION_OF_TRANSLATIONAL_INITIATION | 2.087771 | 0 | 0.00755925 | 1.5931566 | 0.03710938 | 0.12958738 |
| GO_REGULATION_OF_UBIQUITIN_PROTEIN_LIGASE_ACTIVITY | 1.6895868 | 0.02401747 | 0.06201919 | 1.6752465 | 0.02626263 | 0.08670554 |
| GO_REGULATION_OF_VIRAL_TRANSCRIPTION | 1.8461814 | 0.00806452 | 0.02617354 | 1.5137056 | 0.03731343 | 0.18946102 |
| GO_RELEASE_OF_CYTOCHROME_C_FROM_MITOCHONDRIA | 1.515947 | 0.03144654 | 0.15263557 | 1.8209994 | 0 | 0.03319371 |
| GO_REPLICATION_FORK | 1.8122905 | 0.02212389 | 0.03094333 | 1.4650244 | 0.1242485 | 0.22869107 |
| GO_REPLISOME | 1.7041947 | 0.04595186 | 0.05727156 | 1.6288751 | 0.043222 | 0.10929654 |
| GO_RESPIRASOME | 1.8450825 | 0.01282051 | 0.02624841 | 2.0188649 | 0 | 0.00884024 |
| GO_RESPIRATORY_CHAIN_COMPLEX | 1.8119128 | 0.01528384 | 0.03085884 | 1.9990555 | 0 | 0.01032854 |
| GO_RESPIRATORY_ELECTRON_TRANSPORT_CHAIN | 1.8547392 | 0.01766004 | 0.02506167 | 2.0251155 | 0 | 0.00849537 |
| GO_RIBONUCLEASE_ACTIVITY | 1.528741 | 0.05052632 | 0.14467703 | 1.8088295 | 0.00806452 | 0.03696651 |
| GO_RIBONUCLEOPROTEIN_COMPLEX_BINDING | 2.1829453 | 0 | 0.00359299 | 1.8032855 | 0.01162791 | 0.03790001 |
| GO_RIBONUCLEOPROTEIN_COMPLEX_BIOGENESIS | 2.342016 | 0 | 0.00246015 | 2.14659 | 0.00203666 | 0.00738738 |
| GO_RIBONUCLEOPROTEIN_COMPLEX_SUBUNIT_ORGANIZATION | 2.3244767 | 0.00210084 | 0.00224505 | 2.0794508 | 0.00396825 | 0.0067701 |
| GO_RIBONUCLEOSIDE_MONOPHOSPHATE_BIOSYNTHETIC_PROCESS | 1.9671965 | 0 | 0.01422098 | 1.9154685 | 0.00619835 | 0.01632471 |
| GO_RIBONUCLEOSIDE_TRIPHOSPHATE_BIOSYNTHETIC_PROCESS | 1.6747597 | 0.0375 | 0.0684026 | 1.8545575 | 0.00199601 | 0.02560109 |
| GO_RIBOSOMAL_LARGE_SUBUNIT_ASSEMBLY | 1.9034191 | 0 | 0.01844522 | 2.0463142 | 0 | 0.0075476 |
| GO_RIBOSOMAL_LARGE_SUBUNIT_BIOGENESIS | 2.065284 | 0 | 0.00828253 | 2.1434653 | 0 | 0.00734904 |
| GO_RIBOSOMAL_SMALL_SUBUNIT_ASSEMBLY | 1.8340234 | 0.00403226 | 0.02764836 | 1.815332 | 0.00614754 | 0.03491417 |
| GO_RIBOSOMAL_SMALL_SUBUNIT_BIOGENESIS | 2.1569118 | 0 | 0.00436729 | 2.049065 | 0.00205761 | 0.00748552 |
| GO_RIBOSOMAL_SUBUNIT | 2.0386953 | 0.00201207 | 0.01100966 | 2.136396 | 0 | 0.00609175 |
| GO_RIBOSOME | 2.0322864 | 0.00613497 | 0.01093558 | 2.195167 | 0 | 0.00630983 |
| GO_RIBOSOME_ASSEMBLY | 2.1010652 | 0 | 0.00741616 | 2.1297524 | 0 | 0.00553071 |
| GO_RIBOSOME_BINDING | 2.00544 | 0 | 0.01306835 | 2.193787 | 0.00204082 | 0.00574794 |
| GO_RIBOSOME_BIOGENESIS | 2.2326105 | 0 | 0.00320903 | 1.6607826 | 0.03726708 | 0.0925424 |
| GO_RNA_3_END_PROCESSING | 2.4100187 | 0 | 0.00530343 | 1.8244423 | 0.0105042 | 0.03261284 |
| GO_RNA_5_END_PROCESSING | 1.6780379 | 0.02760085 | 0.06712114 | 1.4437112 | 0.08230453 | 0.2454998 |
| GO_RNA_CAP_BINDING | 1.9230462 | 0.00401606 | 0.01686891 | 1.9680464 | 0.00414079 | 0.01195906 |
| GO_RNA_CAPPING | 2.1838481 | 0.002079 | 0.00372528 | 2.181606 | 0.00203666 | 0.0052022 |
| GO_RNA_CATABOLIC_PROCESS | 2.2558725 | 0 | 0.00346842 | 1.5499675 | 0.07476635 | 0.15946089 |
| GO_RNA_DEPENDENT_DNA_BIOSYNTHETIC_PROCESS | 2.2570162 | 0 | 0.00357241 | 1.5115525 | 0.10816327 | 0.1899184 |
| GO_RNA_EXPORT_FROM_NUCLEUS | 2.3827388 | 0.00223214 | 0.00463309 | 1.6152083 | 0.05273438 | 0.11611784 |

| | | | | | | |
|---|---|---|---|---|---|---|
| GO_RNA_METHYLATION | 1.7143462 | 0.02540416 | 0.05418176 | 1.6356089 | 0.036 | 0.1062534 |
| GO_RNA_METHYLTRANSFERASE_ACTIVITY | 1.805025 | 0.02022472 | 0.03184271 | 1.6506046 | 0.04615385 | 0.09772714 |
| GO_RNA_MODIFICATION | 1.900166 | 0.00451467 | 0.01866726 | 1.9891791 | 0.00195695 | 0.01111736 |
| GO_RNA_PHOSPHODIESTER_BOND_HYDROLYSIS | 1.7295674 | 0.02536998 | 0.04956748 | 1.8741207 | 0.00410678 | 0.02260733 |
| GO_RNA_PHOSPHODIESTER_BOND_HYDROLYSIS_ENDONUCLEOLYTIC | 1.5137452 | 0.06105263 | 0.15431039 | 2.0569396 | 0.00215054 | 0.00757445 |
| GO_RNA_POLYMERASE_ACTIVITY | 2.0828018 | 0 | 0.00780415 | 2.0072987 | 0.00404858 | 0.00986767 |
| GO_RNA_POLYMERASE_COMPLEX | 2.3010607 | 0 | 0.00283624 | 1.7377465 | 0.02615694 | 0.05830067 |
| GO_RNA_POLYMERASE_II_CORE_COMPLEX | 1.7821238 | 0.01214575 | 0.03590388 | 1.639473 | 0.01464435 | 0.10400248 |
| GO_RNA_POLYMERASE_II_HOLOENZYME | 2.3081698 | 0 | 0.0029891 | 1.4884511 | 0.06185567 | 0.20789944 |
| GO_RNA_POLYMERASE_III_ACTIVITY | 1.9981948 | 0.00211417 | 0.01284149 | 1.9302332 | 0 | 0.01511183 |
| GO_RNA_POLYMERASE_III_COMPLEX | 1.9933542 | 0 | 0.01314387 | 1.9324027 | 0 | 0.01497538 |
| GO_RNA_SPLICING | 2.2385137 | 0.00450451 | 0.00325312 | 1.7067628 | 0.02620968 | 0.07237629 |
| GO_RNA_SPLICING_VIA_TRANSESTERIFICATION_REACTIONS | 2.265842 | 0.00445434 | 0.00360919 | 1.7859882 | 0.01848049 | 0.04290085 |
| GO_ROUGH_ENDOPLASMIC_RETICULUM | 1.4393644 | 0.04511278 | 0.21404195 | 1.6498958 | 0.014862 | 0.09794338 |
| GO_ROUGH_ENDOPLASMIC_RETICULUM_MEMBRANE | 1.4561732 | 0.05996132 | 0.20154174 | 1.5950701 | 0.04268293 | 0.12864982 |
| GO_RRNA_BINDING | 2.074625 | 0.00207039 | 0.00803262 | 2.055593 | 0 | 0.00753138 |
| GO_RRNA_CONTAINING_RIBONUCLEOPROTEIN_COMPLEX_EXPORT_FROM_NUCLEUS | 1.7955228 | 0 | 0.03354594 | 1.7009948 | 0.02539063 | 0.07421109 |
| GO_RRNA_METABOLIC_PROCESS | 2.2296433 | 0 | 0.00310648 | 2.1830046 | 0.00205761 | 0.00602796 |
| GO_RRNA_METHYLATION | 1.6429317 | 0.02721088 | 0.08089889 | 1.6347203 | 0.03067485 | 0.10656327 |
| GO_RRNA_MODIFICATION | 1.8268757 | 0.01382489 | 0.02822601 | 1.7711686 | 0.01204819 | 0.04754359 |
| GO_RRNA_TRANSCRIPTION | 1.7976512 | 0.02004008 | 0.03318499 | 1.7291468 | 0.01778656 | 0.06226675 |
| GO_S_ADENOSYLMETHIONINE_DEPENDENT_METHYLTRANSFERASE_ACTIVITY | 1.6776258 | 0.02863436 | 0.06722403 | 1.5580345 | 0.04492188 | 0.1535955 |
| GO_SCF_DEPENDENT_PROTEASOMAL_UBIQUITIN_DEPENDENT_PROTEIN_CATABOLIC_PROCESS | 2.0352366 | 0.00205339 | 0.01094633 | 2.0775027 | 0 | 0.00667405 |
| GO_SINGLE_STRANDED_DNA_BINDING | 2.1147523 | 0 | 0.00652656 | 1.5516444 | 0.06496063 | 0.15822425 |
| GO_SM_LIKE_PROTEIN_FAMILY_COMPLEX | 2.1104994 | 0 | 0.00679274 | 2.0873775 | 0 | 0.00695776 |
| GO_SMALL_NUCLEOLAR_RIBONUCLEOPROTEIN_COMPLEX | 2.0710263 | 0 | 0.00821773 | 1.993822 | 0.002079 | 0.0106456 |
| GO_SMALL_RIBOSOMAL_SUBUNIT | 1.9799579 | 0.00398406 | 0.01308186 | 2.0819838 | 0 | 0.00684024 |
| GO_SMALL_SUBUNIT_PROCESSOME | 1.8875377 | 0.00809717 | 0.02011306 | 1.9758158 | 0.00201613 | 0.01176027 |
| GO_SMN_SM_PROTEIN_COMPLEX | 1.8915982 | 0.00197239 | 0.01968319 | 1.9187812 | 0 | 0.01585905 |
| GO_SNORNA_BINDING | 1.9500047 | 0 | 0.01540676 | 1.8725909 | 0.00403226 | 0.02282771 |
| GO_SNRNA_3_END_PROCESSING | 1.7757419 | 0.02145923 | 0.03725673 | 1.9688714 | 0 | 0.01206847 |
| GO_SNRNA_BINDING | 1.8933254 | 0.00838574 | 0.01952337 | 1.7069849 | 0.02526316 | 0.07252984 |
| GO_SNRNA_PROCESSING | 1.7720416 | 0.02202643 | 0.03775061 | 2.0147378 | 0 | 0.00918617 |
| GO_SPLICEOSOMAL_COMPLEX | 2.1985698 | 0.00225734 | 0.00382255 | 1.8678104 | 0.01980198 | 0.02343067 |
| GO_SPLICEOSOMAL_SNRNP_ASSEMBLY | 1.9224453 | 0.00614754 | 0.01686125 | 2.1564794 | 0 | 0.0070151 |
| GO_SPLICEOSOMAL_TRI_SNRNP_COMPLEX | 1.9164953 | 0.00419287 | 0.01730064 | 2.063102 | 0 | 0.00720208 |
| GO_STRUCTURAL_CONSTITUENT_OF_RIBOSOME | 1.9983335 | 0.00205339 | 0.01294077 | 2.0732412 | 0 | 0.00672119 |
| GO_TELOMERASE_HOLOENZYME_COMPLEX | 1.9196061 | 0 | 0.01693326 | 1.6810842 | 0.04008016 | 0.08395609 |
| GO_TELOMERASE_RNA_BINDING | 1.8115909 | 0.00643777 | 0.0308517 | 1.6261655 | 0.05367793 | 0.11049009 |
| GO_TELOMERASE_RNA_LOCALIZATION | 1.7837712 | 0.0101833 | 0.03553075 | 2.016985 | 0 | 0.00900075 |
| GO_TELOMERE_MAINTENANCE_VIA_SEMI_CONSERVATIVE_REPLICATION | 1.8012161 | 0.00643777 | 0.03245752 | 1.8604969 | 0.00601202 | 0.02433595 |
| GO_TELOMERE_MAINTENANCE_VIA_TELOMERE_LENGTHENING | 2.239983 | 0 | 0.00342434 | 1.4361181 | 0.10364683 | 0.24937233 |
| GO_TELOMERE_ORGANIZATION | 2.1649547 | 0 | 0.00434309 | 1.5130447 | 0.08840864 | 0.18869029 |
| GO_TERMINATION_OF_RNA_POLYMERASE_I_TRANSCRIPTION | 1.9154474 | 0.00652174 | 0.01738848 | 1.9417058 | 0.00795229 | 0.01374341 |
| GO_TERMINATION_OF_RNA_POLYMERASE_II_TRANSCRIPTION | 1.9812268 | 0.00617284 | 0.0130393 | 1.504068 | 0.0797546 | 0.19489753 |
| GO_TETRAPYRROLE_BIOSYNTHETIC_PROCESS | 1.4077245 | 0.16075157 | 0.24592766 | 1.6603111 | 0.01632653 | 0.09255717 |
| GO_THREONINE_TYPE_PEPTIDASE_ACTIVITY | 1.6828439 | 0.01048218 | 0.06504586 | 1.827414 | 0.00219298 | 0.03188933 |
| GO_TRANSCRIPTION_BY_RNA_POLYMERASE_I | 2.0015345 | 0.00860215 | 0.01291513 | 1.6136562 | 0.05671078 | 0.11629781 |
| GO_TRANSCRIPTION_BY_RNA_POLYMERASE_III | 1.8672941 | 0.01035197 | 0.02297067 | 1.7028646 | 0.00994036 | 0.07402844 |
| GO_TRANSCRIPTION_COUPLED_NUCLEOTIDE_EXCISION_REPAIR | 2.1266692 | 0 | 0.00617799 | 2.0285935 | 0.002 | 0.00819373 |
| GO_TRANSCRIPTION_ELONGATION_FROM_RNA_POLYMERASE_I_PROMOTER | 1.9243308 | 0.00668151 | 0.01696481 | 1.9507233 | 0.00813008 | 0.01300457 |
| GO_TRANSCRIPTION_ELONGATION_FROM_RNA_POLYMERASE_II_PROMOTER | 2.2818427 | 0 | 0.00306753 | 1.4549658 | 0.0734127 | 0.2381759 |
| GO_TRANSCRIPTION_INITIATION_FROM_RNA_POLYMERASE_I_PROMOTER | 1.8296685 | 0.00898876 | 0.02784528 | 2.0398884 | 0.0039604 | 0.00777261 |
| GO_TRANSCRIPTION_PREINITIATION_COMPLEX_ASSEMBLY | 1.7708912 | 0.0251046 | 0.03806444 | 1.5190939 | 0.04347826 | 0.18398243 |
| GO_TRANSFERASE_ACTIVITY_TRANSFERRING_ONE_CARBON_GROUPS | 1.5627588 | 0.04068523 | 0.122158 | 1.5795317 | 0.04048583 | 0.13895282 |
| GO_TRANSLATION_ELONGATION_FACTOR_ACTIVITY | 1.6362388 | 0.02906977 | 0.08406939 | 1.8676561 | 0.00421053 | 0.02334474 |
| GO_TRANSLATION_FACTOR_ACTIVITY_RNA_BINDING | 2.0785854 | 0.00401606 | 0.00795049 | 1.9406415 | 0.00585938 | 0.01378258 |
| GO_TRANSLATION_INITIATION_FACTOR_ACTIVITY | 2.1172335 | 0 | 0.0066128 | 1.9974966 | 0.00403226 | 0.01038237 |
| GO_TRANSLATION_INITIATION_FACTOR_BINDING | 1.6316019 | 0.03612167 | 0.08627068 | 1.9597777 | 0 | 0.01220353 |
| GO_TRANSLATION_PREINITIATION_COMPLEX | 1.7974648 | 0 | 0.03315038 | 1.8693888 | 0.00788955 | 0.0231517 |
| GO_TRANSLATION_REGULATOR_ACTIVITY | 2.017788 | 0.00201613 | 0.01230293 | 1.906318 | 0.00994036 | 0.01767689 |
| GO_TRANSLATION_REGULATOR_ACTIVITY_NUCLEIC_ACID_BINDING | 1.9776208 | 0.0020202 | 0.01341094 | 1.9532908 | 0.00801603 | 0.01277603 |
| GO_TRANSLATIONAL_ELONGATION | 2.159396 | 0 | 0.00438025 | 2.1327746 | 0 | 0.0060941 |
| GO_TRANSLATIONAL_INITIATION | 2.1869361 | 0.00207039 | 0.00359186 | 2.1533942 | 0.00207039 | 0.00707796 |
| GO_TRANSLATIONAL_TERMINATION | 2.1199782 | 0 | 0.00652231 | 2.1174788 | 0 | 0.00563037 |
| GO_TRANSLESION_SYNTHESIS | 1.9241294 | 0.00220751 | 0.01690564 | 1.4962076 | 0.07171315 | 0.20155528 |
| GO_TRICARBOXYLIC_ACID_CYCLE | 1.5224681 | 0.12184874 | 0.14799733 | 1.5640254 | 0.06868687 | 0.14931361 |
| GO_TRNA_5_END_PROCESSING | 1.7466893 | 0.00425532 | 0.04470521 | 1.9279096 | 0 | 0.01511557 |
| GO_TRNA_BINDING | 1.9615257 | 0.00824742 | 0.01464234 | 1.9188716 | 0.00595238 | 0.01594345 |
| GO_TRNA_METABOLIC_PROCESS | 2.0974853 | 0.00438597 | 0.00728619 | 2.1320405 | 0.00200401 | 0.00593375 |
| GO_TRNA_METHYLATION | 1.5490136 | 0.08278867 | 0.12976718 | 1.6142449 | 0.04581673 | 0.11652426 |
| GO_TRNA_MODIFICATION | 1.9141709 | 0.01079914 | 0.01740481 | 1.9248532 | 0.00400802 | 0.01546901 |
| GO_TRNA_PROCESSING | 2.0039368 | 0.00894855 | 0.01290552 | 2.0912657 | 0.00196464 | 0.0069179 |
| GO_TRNA_SPECIFIC_RIBONUCLEASE_ACTIVITY | 1.7694962 | 0.01079914 | 0.0384407 | 1.91 | 0 | 0.01711638 |
| GO_TRNA_WOBBLE_BASE_MODIFICATION | 1.569126 | 0.0371134 | 0.11822939 | 1.6738384 | 0.03162056 | 0.08705994 |
| GO_U1_SNRNP | 1.5857756 | 0.05776893 | 0.10958181 | 1.6848361 | 0.01584159 | 0.0818421 |
| GO_U12_TYPE_SPLICEOSOMAL_COMPLEX | 1.9263049 | 0.0021322 | 0.01709877 | 1.7385461 | 0.02296451 | 0.05837194 |
| GO_U2_SNRNP | 1.939954 | 0 | 0.01634624 | 1.7678696 | 0.01 | 0.04810723 |
| GO_U2_TYPE_CATALYTIC_STEP_2_SPLICEOSOME | 1.9381659 | 0 | 0.01637241 | 1.8383919 | 0.00806452 | 0.02971813 |
| GO_U2_TYPE_SPLICEOSOMAL_COMPLEX | 2.0664234 | 0.00436681 | 0.00841384 | 1.974765 | 0.00819672 | 0.01184044 |
| GO_U5_SNRNP | 1.788486 | 0.01642711 | 0.03436942 | 1.8335066 | 0 | 0.03073417 |
| GO_UBIQUITIN_PROTEIN_TRANSFERASE_REGULATOR_ACTIVITY | 1.7168332 | 0.0041841 | 0.05340308 | 1.7195828 | 0.0203666 | 0.06634796 |
| GO_UNFOLDED_PROTEIN_BINDING | 1.9328228 | 0.00618557 | 0.01675258 | 1.9711452 | 0.0040568 | 0.01198325 |
| GO_VIRAL_GENE_EXPRESSION | 2.235821 | 0 | 0.00321955 | 2.1392493 | 0.00406504 | 0.00701159 |
| HALLMARK_DNA_REPAIR | 2.2431881 | 0 | 0 | 2.1335196 | 0 | 0.00287088 |
| HALLMARK_E2F_TARGETS | 1.8357594 | 0 | 0.0158998 | 1.673235 | 0.06412826 | 0.07866046 |
| HALLMARK_MTORC1_SIGNALING | 1.948551 | 0.00211417 | 0.00697068 | 1.5586147 | 0.06707317 | 0.11608031 |
| HALLMARK_MYC_TARGETS_V1 | 2.0653164 | 0 | 0.00245984 | 2.1271436 | 0 | 0.0018283 |
| HALLMARK_MYC_TARGETS_V2 | 1.9752315 | 0 | 0.00638954 | 2.0013602 | 0 | 0.00528966 |
| HALLMARK_OXIDATIVE_PHOSPHORYLATION | 1.8664356 | 0.00970874 | 0.01333482 | 2.0969865 | 0 | 0.0020897 |
| HALLMARK_UNFOLDED_PROTEIN_RESPONSE | 2.0384197 | 0 | 0.00302806 | 1.7225155 | 0.0259481 | 0.06410048 |
| HALLMARK_UV_RESPONSE_UP | 1.472364 | 0.05060729 | 0.19898874 | 1.53753 | 0.02070393 | 0.1163805 |
| KEGG_ALZHEIMERS_DISEASE | 1.5431873 | 0.086 | 0.17683585 | 1.9762418 | 0 | 0.01539847 |
| KEGG_AMINOACYL_TRNA_BIOSYNTHESIS | 1.882898 | 0.00829876 | 0.02469181 | 1.8239313 | 0.01244813 | 0.02628775 |
| KEGG_BASE_EXCISION_REPAIR | 1.6271341 | 0.04989605 | 0.11365747 | 1.8273188 | 0 | 0.02658051 |
| KEGG_DNA_REPLICATION | 1.6245027 | 0.03571429 | 0.11010298 | 1.9397784 | 0.0020202 | 0.01472231 |
| KEGG_HOMOLOGOUS_RECOMBINATION | 1.649009 | 0.0260521 | 0.10039916 | 1.6434948 | 0.05048544 | 0.10267932 |
| KEGG_HUNTINGTONS_DISEASE | 1.8896232 | 0.01419878 | 0.03001381 | 1.9826726 | 0 | 0.01739469 |
| KEGG_MISMATCH_REPAIR | 1.7011684 | 0.0125 | 0.07942785 | 1.7448819 | 0.02254098 | 0.04997789 |
| KEGG_NUCLEOTIDE_EXCISION_REPAIR | 1.877586 | 0.01473684 | 0.02341295 | 1.9458975 | 0.00198413 | 0.01564782 |
| KEGG_ONE_CARBON_POOL_BY_FOLATE | 1.4823636 | 0.07784431 | 0.22015722 | 1.5238873 | 0.04536489 | 0.1988253 |
| KEGG_OXIDATIVE_PHOSPHORYLATION | 1.8853046 | 0.02857143 | 0.02762242 | 2.0368764 | 0 | 0.02402001 |
| KEGG_PARKINSONS_DISEASE | 1.6665127 | 0.07480315 | 0.09438139 | 2.0078273 | 0 | 0.01494241 |
| KEGG_PROTEASOME | 1.7589918 | 0 | 0.04971591 | 1.8702842 | 0 | 0.01861646 |
| KEGG_PROTEIN_EXPORT | 1.6999947 | 0.02941177 | 0.07536143 | 1.8407952 | 0.01037344 | 0.02449707 |
| KEGG_PYRIMIDINE_METABOLISM | 1.9321352 | 0.00409836 | 0.01964345 | 2.0342622 | 0 | 0.01262364 |
| KEGG_RIBOSOME | 1.8032627 | 0.00369686 | 0.03861523 | 1.9055556 | 0 | 0.01480187 |
| KEGG_RNA_DEGRADATION | 2.096173 | 0.00211417 | 0.01113524 | 1.6098111 | 0.05631068 | 0.12464078 |
| KEGG_RNA_POLYMERASE | 1.976757 | 0.00209205 | 0.01972652 | 1.9135367 | 0 | 0.01533381 |
| KEGG_SPLICEOSOME | 2.1573892 | 0.00636943 | 0.0102908 | 1.9688283 | 0.00792079 | 0.01373643 |
| MUHAR_MYC | 2.056677 | 0 | 0 | 2.0271058 | 0.00396825 | 0.00396825 |
| REACTOME_ABC_TRANSPORTER_DISORDERS | 1.5155689 | 0.06144068 | 0.11790481 | 2.0533473 | 0 | 0.00651571 |
| REACTOME_ABORTIVE_ELONGATION_OF_HIV_1_TRANSCRIPT_IN_THE_ABSENCE_OF_TAT | 2.0941167 | 0 | 0.00455345 | 1.8280381 | 0.00603622 | 0.02439161 |
| REACTOME_ACTIVATION_OF_APC_C_AND_APC_C:CDC20_MEDIATED_DEGRADATION_OF_MITOTIC_PROTEINS | 1.8943316 | 0 | 0.0140302 | 2.0254102 | 0 | 0.00669617 |
| REACTOME_ACTIVATION_OF_ATR_IN_RESPONSE_TO_REPLICATION_STRESS | 1.7317466 | 0.00674157 | 0.03799924 | 1.584648 | 0.06889764 | 0.096573 |
| REACTOME_ACTIVATION_OF_NF_KAPPAB_IN_B_CELLS | 1.7859186 | 0.01649485 | 0.02643907 | 1.8880492 | 0.01388889 | 0.01550386 |
| REACTOME_ACTIVATION_OF_THE_MRNA_UPON_BINDING_OF_THE_CAP_BINDING_COMPLEX_AND_EIFS_AND_SUBSEQUENT_BINDING_TO_43S | 1.9022554 | 0.00201613 | 0.01364553 | 1.961412 | 0 | 0.00881232 |
| REACTOME_ACTIVATION_OF_THE_PRE_REPLICATIVE_COMPLEX | 1.6589166 | 0.01565995 | 0.05832152 | 1.7225527 | 0.03543307 | 0.0453626 |
| REACTOME_ANTIGEN_PROCESSING_CROSS_PRESENTATION | 1.5519149 | 0.08146639 | 0.09980982 | 1.784507 | 0.02620968 | 0.03240068 |
| REACTOME_ANTIGEN_PROCESSING:_UBIQUITINATION_PROTEASOME_DEGRADATION | 1.84013 | 0.00700935 | 0.01891318 | 1.390512 | 0.05686275 | 0.22265686 |
| REACTOME_APC_C:CDC20_MEDIATED_DEGRADATION_OF_CYCLIN_B | 1.8337784 | 0 | 0.01981006 | 1.8592141 | 0 | 0.02035414 |
| REACTOME_APC_C:CDH1_MEDIATED_DEGRADATION_OF_CDC20_AND_OTHER_APC_C:CDH1_TARGETED_PROTEINS_IN_LATE_MITOSIS_EARLY_G1 | 1.9559425 | 0 | 0.00965798 | 2.0407665 | 0 | 0.00640434 |
| REACTOME_APC_CDC20_MEDIATED_DEGRADATION_OF_NEK2A | 1.849929 | 0 | 0.018104 | 1.7783568 | 0.0189394 | 0.03362412 |
| REACTOME_ASSEMBLY_OF_THE_PRE_REPLICATIVE_COMPLEX | 1.8740636 | 0.0021692 | 0.01550704 | 2.0394232 | 0 | 0.00629452 |
| REACTOME_ASSOCIATION_OF_TRIC_CCT_WITH_TARGET_PROTEINS_DURING_BIOSYNTHESIS | 1.804524 | 0.01535088 | 0.02352658 | 1.7065666 | 0.01444043 | 0.05049564 |
| REACTOME_ASYMMETRIC_LOCALIZATION_OF_PCP_PROTEINS | 1.7341 | 0.04928131 | 0.03790209 | 1.9812587 | 0.0020202 | 0.00777195 |
| REACTOME_ATF4_ACTIVATES_GENES_IN_RESPONSE_TO_ENDOPLASMIC_RETICULUM_STRESS | 1.7504379 | 0.02857143 | 0.03422477 | 1.9474682 | 0 | 0.00957473 |
| REACTOME_AUF1_HNRNP_D0_BINDS_AND_DESTABILIZES_MRNA | 1.8840169 | 0 | 0.01498933 | 2.0110261 | 0 | 0.00711973 |
| REACTOME_AUTOPHAGY | 1.568769 | 0.05579399 | 0.0925246 | 1.6092652 | 0.02390438 | 0.0866859 |
| REACTOME_BASE_EXCISION_REPAIR | 1.7733467 | 0.01360544 | 0.02916792 | 1.6406552 | 0.04056795 | 0.07311789 |
| REACTOME_BUDDING_AND_MATURATION_OF_HIV_VIRION | 1.333582 | 0.17805383 | 0.23225749 | 1.5974009 | 0.05656566 | 0.0904952 |
| REACTOME_BUTYRATE_RESPONSE_FACTOR_1_BRF1_BINDS_AND_DESTABILIZES_MRNA | 1.5610219 | 0.07727273 | 0.09639601 | 1.7372025 | 0.01744186 | 0.04198539 |
| REACTOME_C_TYPE_LECTIN_RECEPTORS_CLRS | 1.345016 | 0.16044776 | 0.22483985 | 1.5953227 | 0.04545455 | 0.09075376 |
| REACTOME_CDK_MEDIATED_PHOSPHORYLATION_AND_REMOVAL_OF_CDC6 | 2.0102255 | 0 | 0.00648616 | 2.061537 | 0 | 0.0068883 |
| REACTOME_CELL_CYCLE_CHECKPOINTS | 1.9064995 | 0.00223714 | 0.01350112 | 1.6681932 | 0.0665362 | 0.06261517 |
| REACTOME_CELL_CYCLE_MITOTIC | 1.9226395 | 0.00451467 | 0.01226561 | 1.5837086 | 0.07827789 | 0.0966064 |
| REACTOME_CELLULAR_RESPONSE_TO_HYPOXIA | 2.038795 | 0.00210971 | 0.00526397 | 1.9839809 | 0.00403226 | 0.00769368 |

| | | | | | | |
|---|---|---|---|---|---|---|
| REACTOME_CELLULAR_RESPONSES_TO_EXTERNAL_STIMULI | 2.0772338 | 0 | 0.00471376 | 1.6662755 | 0.00829876 | 0.06310565 |
| REACTOME_CELLULAR_RESPONSES_TO_STRESS | 2.0289843 | 0.0021692 | 0.00533264 | 1.6045281 | 0.02053388 | 0.08849589 |
| REACTOME_CHROMOSOME_MAINTENANCE | 1.779686 | 0.00450451 | 0.02760413 | 1.6951572 | 0.04106776 | 0.05450426 |
| REACTOME_CITRIC_ACID_CYCLE_TCA_CYCLE | 1.685309 | 0.04237288 | 0.05091537 | 1.5763233 | 0.05175984 | 0.09951824 |
| REACTOME_CLEC7A_DECTIN_1_SIGNALING | 1.866092 | 0.00626305 | 0.01640476 | 1.930812 | 0 | 0.01077784 |
| REACTOME_CLEC7A_DECTIN_1_SIGNALING | 1.866092 | 0.00626305 | 0.01640476 | 1.7751215 | 0.02366864 | 0.03420201 |
| REACTOME_COMPLEX_I_BIOGENESIS | 1.8396434 | 0.01545254 | 0.01889371 | 1.5765611 | 0.03787879 | 0.0999051 |
| REACTOME_COOPERATION_OF_PREFOLDIN_AND_TRIC_CCT_IN_ACTIN_AND_TUBULIN_FOLDING | 1.8571782 | 0.00413223 | 0.01725428 | 2.056853 | 0 | 0.00648126 |
| REACTOME_CROSS_PRESENTATION_OF_SOLUBLE_EXOGENOUS_ANTIGENS_ENDOSOMES | 1.7670207 | 0.00650759 | 0.03007402 | 1.9675492 | 0.00402415 | 0.00857848 |
| REACTOME_CYCLIN_A_B1_B2_ASSOCIATED_EVENTS_DURING_G2_M_TRANSITION | 1.5748835 | 0.04625551 | 0.09042875 | 1.6800522 | 0.04296875 | 0.06040163 |
| REACTOME_CYCLIN_A:CDK2_ASSOCIATED_EVENTS_AT_S_PHASE_ENTRY | 2.0822902 | 0 | 0.00469404 | 1.933779 | 0.00581395 | 0.01060398 |
| REACTOME_CYTOSOLIC_SENSORS_OF_PATHOGEN_ASSOCIATED_DNA | 1.8536217 | 0.00652174 | 0.01775036 | 1.4133667 | 0.05747126 | 0.20676237 |
| REACTOME_CYTOSOLIC_TRNA_AMINOACYLATION | 1.714602 | 0.00425532 | 0.04235866 | 1.946163 | 0 | 0.00945966 |
| REACTOME_DEADENYLATION_DEPENDENT_MRNA_DECAY | 1.9930372 | 0.00689655 | 0.00719349 | 1.7966589 | 0.00965251 | 0.03098843 |
| REACTOME_DECTIN_1_MEDIATED_NONCANONICAL_NF_KB_SIGNALING | 1.9134146 | 0.0040568 | 0.01277288 | 2.0121722 | 0.00598802 | 0.00712975 |
| REACTOME_DEFECTIVE_CFTR_CAUSES_CYSTIC_FIBROSIS | 1.8812842 | 0.00819672 | 0.01507027 | 1.9932431 | 0 | 0.00725469 |
| REACTOME_DEGRADATION_OF_AXIN | 1.7047421 | 0.01914894 | 0.04568336 | 1.9265834 | 0.00398406 | 0.01105137 |
| REACTOME_DEGRADATION_OF_BETA_CATENIN_BY_THE_DESTRUCTION_COMPLEX | 1.7323086 | 0.03893443 | 0.03799569 | 2.021451 | 0.00200803 | 0.00650696 |
| REACTOME_DEGRADATION_OF_DVL | 1.9369954 | 0 | 0.0109852 | 2.0020728 | 0 | 0.00718792 |
| REACTOME_DEGRADATION_OF_GLI1_BY_THE_PROTEASOME | 1.8235619 | 0.00208768 | 0.02138371 | 2.0023396 | 0.00198807 | 0.00729457 |
| REACTOME_DISEASES_ASSOCIATED_WITH_N_GLYCOSYLATION_OF_PROTEINS | 1.6708344 | 0.02826087 | 0.05465856 | 1.403984 | 0.12684989 | 0.21391825 |
| REACTOME_DNA_DAMAGE_BYPASS | 1.8984023 | 0.01133787 | 0.0138251 | 1.6092749 | 0.03653846 | 0.0871622 |
| REACTOME_DNA_DAMAGE_RECOGNITION_IN_GG_NER | 2.0199854 | 0.00453515 | 0.00587098 | 1.8698465 | 0.0056926 | 0.0185661 |
| REACTOME_DNA_REPAIR | 2.0679429 | 0.002331 | 0.00488687 | 1.5261482 | 0.07251909 | 0.128744 |
| REACTOME_DNA_REPLICATION | 1.8128762 | 0.00456621 | 0.02275313 | 2.1111357 | 0 | 0.00476373 |
| REACTOME_DNA_REPLICATION_PRE_INITIATION | 1.8186159 | 0.00449438 | 0.0218719 | 2.0219975 | 0 | 0.00666188 |
| REACTOME_DNA_STRAND_ELONGATION | 1.58576 | 0.02721088 | 0.08620298 | 1.7962183 | 0.01547389 | 0.03082844 |
| REACTOME_DOWNSTREAM_SIGNALING_EVENTS_OF_B_CELL_RECEPTOR_BCR | 1.7936503 | 0.02263374 | 0.02515944 | 1.8039687 | 0.02584493 | 0.02979787 |
| REACTOME_DOWNSTREAM_TCR_SIGNALING | 1.4156517 | 0.18181819 | 0.1748493 | 1.7898808 | 0.0254902 | 0.031878 |
| REACTOME_DUAL_INCISION_IN_GG_NER | 1.8893409 | 0.00681818 | 0.01453917 | 1.9275696 | 0.00194553 | 0.01102455 |
| REACTOME_DUAL_INCISION_IN_TC_NER | 2.0519087 | 0 | 0.00524419 | 1.9472895 | 0.0019305 | 0.00948863 |
| REACTOME_E2F_MEDIATED_REGULATION_OF_DNA_REPLICATION | 1.4963439 | 0.12211981 | 0.12783322 | 1.5115738 | 0.07905138 | 0.13666023 |
| REACTOME_ENDOSOMAL_SORTING_COMPLEX_REQUIRED_FOR_TRANSPORT_ESCRT | 1.402968 | 0.13168724 | 0.18276492 | 1.5458133 | 0.06972112 | 0.11579149 |
| REACTOME_EUKARYOTIC_TRANSLATION_INITIATION | 1.8935987 | 0.00205761 | 0.01400914 | 2.0075946 | 0 | 0.00723973 |
| REACTOME_EXTENSION_OF_TELOMERES | 1.6693677 | 0.01830664 | 0.05512565 | 1.9502113 | 0.00192678 | 0.00936918 |
| REACTOME_FANCONI_ANEMIA_PATHWAY | 1.8858042 | 0.00444445 | 0.01493684 | 1.5026183 | 0.08206107 | 0.14036965 |
| REACTOME_FBXL7_DOWN_REGULATES_AURKA_DURING_MITOTIC_ENTRY_AND_IN_EARLY_MITOSIS | 1.884426 | 0 | 0.01510691 | 2.0020697 | 0 | 0.00703498 |
| REACTOME_FGFR2_ALTERNATIVE_SPLICING | 2.0705 | 0.00429185 | 0.00499285 | 1.7710168 | 0.00795229 | 0.03466808 |
| REACTOME_FORMATION_OF_HIV_ELONGATION_COMPLEX_IN_THE_ABSENCE_OF_HIV_TAT | 2.2766817 | 0 | 9.08E-04 | 1.77481 | 0.00395257 | 0.03411728 |
| REACTOME_FORMATION_OF_INCISION_COMPLEX_IN_GG_NER | 2.0931168 | 0.00223714 | 0.00430732 | 1.6649512 | 0.01876173 | 0.06333054 |
| REACTOME_FORMATION_OF_TC_NER_PRE_INCISION_COMPLEX | 2.166558 | 0 | 0.00312782 | 1.9408137 | 0 | 0.00988994 |
| REACTOME_FORMATION_OF_THE_EARLY_ELONGATION_COMPLEX | 2.1862793 | 0 | 0.00212476 | 1.9397168 | 0 | 0.00993919 |
| REACTOME_FORMATION_OF_TUBULIN_FOLDING_INTERMEDIATES_BY_CCT_TRIC | 1.7275188 | 0.02385686 | 0.03879828 | 2.1080213 | 0 | 0.00469781 |
| REACTOME_G1_PHASE | 1.6747853 | 0.01580136 | 0.05385908 | 1.38826 | 0.0965251 | 0.22315574 |
| REACTOME_G1_S_DNA_DAMAGE_CHECKPOINTS | 1.969583 | 0 | 0.00896889 | 2.019934 | 0 | 0.00653659 |
| REACTOME_G2_M_CHECKPOINTS | 1.86807 | 0.00453515 | 0.01626517 | 1.767335 | 0.02647658 | 0.03530783 |
| REACTOME_GAP_FILLING_DNA_REPAIR_SYNTHESIS_AND_LIGATION_IN_GG_NER | 1.6202799 | 0.05287356 | 0.07226455 | 1.8013569 | 0.01181102 | 0.03044556 |
| REACTOME_GENE_AND_PROTEIN_EXPRESSION_BY_JAK_STAT_SIGNALING_AFTER_INTERLEUKIN_12_STIMULATION | 1.7681276 | 0.01082251 | 0.02998463 | 1.5142678 | 0.07645875 | 0.1359078 |
| REACTOME_GLOBAL_GENOME_NUCLEOTIDE_EXCISION_REPAIR_GG_NER | 2.047779 | 0.00234742 | 0.00543588 | 1.8997319 | 0.00191205 | 0.01409247 |
| REACTOME_GLUCOSE_METABOLISM | 1.6745337 | 0.02783726 | 0.05376211 | 1.5066876 | 0.03529412 | 0.1397364 |
| REACTOME_HDR_THROUGH_HOMOLOGOUS_RECOMBINATION_HRR | 1.8148118 | 0.02192983 | 0.02256042 | 1.4290133 | 0.1447619 | 0.19468687 |
| REACTOME_HEDGEHOG_LIGAND_BIOGENESIS | 1.6083832 | 0.06126482 | 0.07701983 | 2.0067043 | 0.00205339 | 0.00710608 |
| REACTOME_HEDGEHOG_OFF_STATE | 1.3765708 | 0.14718615 | 0.20122658 | 1.8193516 | 0.01160542 | 0.02601918 |
| REACTOME_HIV_ELONGATION_ARREST_AND_RECOVERY | 2.1582744 | 0 | 0.00320618 | 1.8459045 | 0.00770713 | 0.02241 |
| REACTOME_HIV_INFECTION | 2.242397 | 0 | 0.00164362 | 1.6626359 | 0.01028807 | 0.06405067 |
| REACTOME_HIV_LIFE_CYCLE | 2.3258238 | 0 | 0.00107082 | 1.9933099 | 0 | 0.00739978 |
| REACTOME_HOST_INTERACTIONS_OF_HIV_FACTORS | 1.9976398 | 0.00212314 | 0.00714514 | 1.7416037 | 0.00932836 | 0.04092439 |
| REACTOME_HSF1_ACTIVATION | 1.5403883 | 0.09421842 | 0.10544926 | 2.0385168 | 0 | 0.00622179 |
| REACTOME_INFECTIOUS_DISEASE | 2.3802943 | 0 | 0.00308723 | 1.8298717 | 0.01939655 | 0.02400171 |
| REACTOME_INFLUENZA_INFECTION | 2.1646502 | 0 | 0.00307655 | 2.1902258 | 0 | 0.00203268 |
| REACTOME_INHIBITION_OF_THE_PROTEOLYTIC_ACTIVITY_OF_APC_C_REQUIRED_FOR_THE_ONSET_OF_ANAPHASE_BY_MITOTIC_SPINDLE_CHECKPOINT_COMPONENTS | 1.7867084 | 0 | 0.02638664 | 1.6245164 | 0.0510397 | 0.08023795 |
| REACTOME_INSERTION_OF_TAIL_ANCHORED_PROTEINS_INTO_THE_ENDOPLASMIC_RETICULUM_MEMBRANE | 1.6032367 | 0.04938272 | 0.07877643 | 1.742589 | 0.01234568 | 0.04084685 |
| REACTOME_INTERACTIONS_OF_REV_WITH_HOST_CELLULAR_PROTEINS | 2.0082417 | 0 | 0.00650114 | 1.742589 | 0.01234568 | 0.04084685 |
| REACTOME_INTERACTIONS_OF_VPR_WITH_HOST_CELLULAR_PROTEINS | 2.0919504 | 0 | 0.00429362 | 1.3698878 | 0.16318786 | 0.23611203 |
| REACTOME_INTERCONVERSION_OF_NUCLEOTIDE_DI_AND_TRIPHOSPHATES | 1.5195963 | 0.06029106 | 0.11589322 | 1.5695513 | 0.0625 | 0.10240564 |
| REACTOME_INTERLEUKIN_1_FAMILY_SIGNALING | 1.8979396 | 0.00622407 | 0.01374058 | 1.4935197 | 0.06986028 | 0.14423707 |
| REACTOME_INTERLEUKIN_1_SIGNALING | 2.0371792 | 0 | 0.00522422 | 1.7122355 | 0.03137255 | 0.04870746 |
| REACTOME_KSRP_KHSRP_BINDS_AND_DESTABILIZES_MRNA | 1.7661648 | 0.01431981 | 0.03019848 | 1.9312353 | 0 | 0.01086584 |
| REACTOME_LAGGING_STRAND_SYNTHESIS | 1.5559177 | 0.05263158 | 0.09838529 | 1.8455666 | 0.0019305 | 0.02230545 |
| REACTOME_M_PHASE | 1.988349 | 0.00223714 | 0.00746962 | 1.5549337 | 0.08076923 | 0.11016733 |
| REACTOME_MACROAUTOPHAGY | 1.5605414 | 0.07526882 | 0.09636527 | 1.6575164 | 0.00988142 | 0.06611259 |
| REACTOME_MAPK6_MAPK4_SIGNALING | 1.9018323 | 0.01518438 | 0.01356729 | 1.8446978 | 0.0185567 | 0.0223082 |
| REACTOME_METABOLISM_OF_AMINO_ACIDS_AND_DERIVATIVES | 1.6660275 | 0.02575107 | 0.05610034 | 2.3036551 | 0 | 0.00140891 |
| REACTOME_METABOLISM_OF_COFACTORS | 1.68201 | 0.01096491 | 0.05198783 | 1.6184752 | 0.03921569 | 0.08242334 |
| REACTOME_METABOLISM_OF_FOLATE_AND_PTERINES | 1.5063604 | 0.06564552 | 0.12242222 | 1.7624753 | 0.00961539 | 0.03598939 |
| REACTOME_METABOLISM_OF_NON_CODING_RNA | 2.1277113 | 0 | 0.00368204 | 1.7324545 | 0.03047619 | 0.04278224 |
| REACTOME_METABOLISM_OF_NUCLEOTIDES | 1.7590241 | 0.00213675 | 0.03215667 | 1.8660555 | 0.00598802 | 0.0190005 |
| REACTOME_METABOLISM_OF_POLYAMINES | 2.0559008 | 0.00214592 | 0.00531063 | 2.0583348 | 0 | 0.00670523 |
| REACTOME_METABOLISM_OF_PORPHYRINS | 1.3290925 | 0.14401622 | 0.2355269 | 1.5007793 | 0.06198347 | 0.14050111 |
| REACTOME_MICRORNA_MIRNA_BIOGENESIS | 1.8335073 | 0.00441501 | 0.01975593 | 1.8533666 | 0.004 | 0.02112982 |
| REACTOME_MISMATCH_REPAIR | 1.692337 | 0.01754386 | 0.04849545 | 1.6684688 | 0.02087287 | 0.06331404 |
| REACTOME_MITOCHONDRIAL_CALCIUM_ION_TRANSPORT | 1.454563 | 0.10183299 | 0.15060389 | 2.027394 | 0 | 0.00691483 |
| REACTOME_MITOCHONDRIAL_PROTEIN_IMPORT | 1.9357613 | 0.00222717 | 0.01101033 | 2.0470297 | 0 | 0.00626167 |
| REACTOME_MITOCHONDRIAL_TRANSLATION | 2.0939116 | 0 | 0.00442696 | 2.0539854 | 0 | 0.0068415 |
| REACTOME_MITOCHONDRIAL_TRNA_AMINOACYLATION | 1.7093207 | 0.01952278 | 0.04408638 | 1.8572376 | 0.00766284 | 0.02051254 |
| REACTOME_MITOPHAGY | 1.4262942 | 0.14254385 | 0.16855194 | 1.750783 | 0.00408163 | 0.0383669 |
| REACTOME_MITOTIC_G1_G1_S_PHASES | 1.8995903 | 0.00443459 | 0.01378556 | 1.9027498 | 0.01171875 | 0.01381581 |
| REACTOME_MITOTIC_G2_G2_M_PHASES | 2.0522914 | 0.00448431 | 0.00533021 | 1.8435581 | 0.00766284 | 0.02219286 |
| REACTOME_MITOTIC_METAPHASE_AND_ANAPHASE | 1.9599552 | 0.00223214 | 0.00950266 | 1.7848799 | 0.0368217 | 0.03254615 |
| REACTOME_MITOTIC_SPINDLE_CHECKPOINT | 1.803724 | 0.00881057 | 0.02356927 | 1.3972945 | 0.1782364 | 0.21770214 |
| REACTOME_MRNA_CAPPING | 2.0564995 | 0 | 0.0053212 | 1.9292984 | 0 | 0.01079751 |
| REACTOME_MRNA_DECAY_BY_3_TO_5_EXORIBONUCLEASE | 1.6946718 | 0.01385681 | 0.04796165 | 1.9324547 | 0.0019802 | 0.01075785 |
| REACTOME_MRNA_SPLICING | 2.203924 | 0 | 0.00192523 | 1.9504136 | 0.00199601 | 0.00944188 |
| REACTOME_MRNA_SPLICING_MINOR_PATHWAY | 2.0965753 | 0 | 0.00457203 | 2.048592 | 0 | 0.00660857 |
| REACTOME_MTORC1_MEDIATED_SIGNALLING | 1.6277181 | 0.03950104 | 0.06923267 | 1.4932532 | 0.04624277 | 0.14374647 |
| REACTOME_NEDDYLATION | 1.8047508 | 0.0212766 | 0.02370463 | 1.7680198 | 0.0020202 | 0.03535459 |
| REACTOME_NEGATIVE_EPIGENETIC_REGULATION_OF_RRNA_EXPRESSION | 1.8460368 | 0.00219298 | 0.01855034 | 1.4037459 | 0.13473684 | 0.21325012 |
| REACTOME_NEGATIVE_REGULATION_OF_NOTCH4_SIGNALING | 1.8516599 | 0 | 0.01788137 | 1.9606618 | 0.00200803 | 0.00873733 |
| REACTOME_NEURODEGENERATIVE_DISEASES | 1.5670327 | 0.06540085 | 0.09319289 | 1.3774531 | 0.13142857 | 0.2311392 |
| REACTOME_NONSENSE_MEDIATED_DECAY_NMD | 2.0609376 | 0 | 0.00510986 | 2.092921 | 0 | 0.00568944 |
| REACTOME_NONSENSE_MEDIATED_DECAY_NMD_INDEPENDENT_OF_THE_EXON_JUNCTION_COMPLEX_EJC | 1.862448 | 0.00404858 | 0.01676564 | 1.983071 | 0 | 0.00770437 |
| REACTOME_NUCLEOBASE_BIOSYNTHESIS | 1.6637809 | 0.03099174 | 0.05662197 | 1.7661344 | 0.00400802 | 0.03546388 |
| REACTOME_NUCLEOTIDE_EXCISION_REPAIR | 2.1506307 | 0 | 0.00346693 | 1.9517094 | 0.00190476 | 0.00931993 |
| REACTOME_NUCLEOTIDE_SALVAGE | 1.7366523 | 0.0021692 | 0.03766616 | 1.6011051 | 0.03703704 | 0.0895169 |
| REACTOME_ORC1_REMOVAL_FROM_CHROMATIN | 1.8660353 | 0 | 0.016304 | 2.0443046 | 0 | 0.00627153 |
| REACTOME_PCNA_DEPENDENT_LONG_PATCH_BASE_EXCISION_REPAIR | 1.6407242 | 0.04026846 | 0.06466235 | 1.8445467 | 0.00383877 | 0.02212824 |
| REACTOME_PCP_CE_PATHWAY | 1.6494004 | 0.05319149 | 0.06112796 | 1.9242328 | 0.00584795 | 0.01113786 |
| REACTOME_PERK_REGULATES_GENE_EXPRESSION | 1.8863239 | 0.01229508 | 0.01496518 | 1.8972747 | 0.00203252 | 0.01433176 |
| REACTOME_PHOSPHORYLATION_OF_THE_APC_C | 1.7700448 | 0 | 0.02972205 | 1.7711407 | 0.01672863 | 0.03490493 |
| REACTOME_POST_CHAPERONIN_TUBULIN_FOLDING_PATHWAY | 1.5847287 | 0.06420233 | 0.08642687 | 1.7952077 | 0.00877193 | 0.03085641 |
| REACTOME_PREFOLDIN_MEDIATED_TRANSFER_OF_SUBSTRATE_TO_CCT_TRIC | 1.8224194 | 0.00425532 | 0.02150116 | 2.0342474 | 0 | 0.00635696 |
| REACTOME_PROCESSING_OF_CAPPED_INTRON_CONTAINING_PRE_MRNA | 2.2620168 | 0 | 0.00126037 | 1.9236974 | 0.0039604 | 0.01108251 |
| REACTOME_PROCESSING_OF_CAPPED_INTRONLESS_PRE_MRNA | 1.9051381 | 0.00223714 | 0.01358957 | 1.5021688 | 0.09386973 | 0.14006568 |
| REACTOME_PROCESSIVE_SYNTHESIS_ON_THE_LAGGING_STRAND | 1.5536127 | 0.04072398 | 0.09912106 | 1.8685979 | 0.00579151 | 0.0186662 |
| REACTOME_PROGRAMMED_CELL_DEATH | 1.8827889 | 0.00842105 | 0.01495911 | 1.5709138 | 0.04961832 | 0.10260267 |
| REACTOME_PROTEIN_FOLDING | 1.5005196 | 0.06170213 | 0.12536396 | 1.8820802 | 0 | 0.01634499 |
| REACTOME_PROTEIN_LOCALIZATION | 1.8311005 | 0.01495727 | 0.02004243 | 2.0310276 | 0.00206612 | 0.00664188 |
| REACTOME_PTEN_REGULATION | 1.8742226 | 0.01301519 | 0.01572336 | 1.7363801 | 0.01171875 | 0.04203285 |
| REACTOME_PURINE_CATABOLISM | 1.5266092 | 0.06485356 | 0.11168794 | 1.5973309 | 0.03968254 | 0.0900433 |
| REACTOME_PYRUVATE_METABOLISM_AND_CITRIC_ACID_TCA_CYCLE | 1.5816333 | 0.06651885 | 0.08791389 | 1.5031377 | 0.06832298 | 0.14058025 |
| REACTOME_RECOGNITION_OF_DNA_DAMAGE_BY_PCNA_CONTAINING_REPLICATION_COMPLEX | 1.7022805 | 0.03363229 | 0.04615493 | 1.7989024 | 0.00984252 | 0.0306144 |
| REACTOME_REGULATION_OF_APOPTOSIS | 1.8427761 | 0 | 0.01865291 | 1.9852704 | 0 | 0.00785487 |
| REACTOME_REGULATION_OF_EXPRESSION_OF_SLITS_AND_ROBOS | 2.2761066 | 0 | 8.17E-04 | 2.1778064 | 0 | 0.00232255 |
| REACTOME_REGULATION_OF_MITOTIC_CELL_CYCLE | 1.8712381 | 0 | 0.01584004 | 2.0261827 | 0.00583658 | 0.00681026 |
| REACTOME_REGULATION_OF_MRNA_STABILITY_BY_PROTEINS_THAT_BIND_AU_RICH_ELEMENTS | 2.0325675 | 0 | 0.00534802 | 1.9977515 | 0.002 | 0.0072903 |
| REACTOME_REGULATION_OF_PTEN_STABILITY_AND_ACTIVITY | 1.9606483 | 0.00208768 | 0.00970154 | 1.9077511 | 0.00816327 | 0.01314709 |
| REACTOME_REGULATION_OF_RAS_BY_GAPS | 2.0455818 | 0 | 0.00546593 | 1.8391595 | 0.01581028 | 0.02297514 |
| REACTOME_REGULATION_OF_RUNX2_EXPRESSION_AND_ACTIVITY | 1.8783762 | 0.02272727 | 0.0153763 | 1.9712842 | 0.00196078 | 0.00862541 |
| REACTOME_REGULATION_OF_RUNX3_EXPRESSION_AND_ACTIVITY | 2.0407257 | 0 | 0.00544684 | 1.9700857 | 0.00197239 | 0.00861888 |
| REACTOME_REGULATION_OF_TP53_ACTIVITY_THROUGH_PHOSPHORYLATION | 1.8969254 | 0.00454545 | 0.01376046 | 1.3716251 | 0.14694656 | 0.23526737 |
| REACTOME_RESOLUTION_OF_ABASIC_SITES_AP_SITES | 1.7337397 | 0.02073733 | 0.03784657 | 1.9672034 | 0 | 0.0084728 |
| REACTOME_RESOLUTION_OF_AP_SITES_VIA_THE_MULTIPLE_NUCLEOTIDE_PATCH_REPLACEMENT_PATHWAY | 1.6941433 | 0.02449889 | 0.04796267 | 1.7930579 | 0.00761905 | 0.03121428 |
| REACTOME_RESPIRATORY_ELECTRON_TRANSPORT | 1.960416 | 0.00867679 | 0.00960106 | 2.0023706 | 0 | 0.00744203 |
| REACTOME_RESPIRATORY_ELECTRON_TRANSPORT_ATP_SYNTHESIS_BY_CHEMIOSMOTIC_COUPLING_AND_HEAT_PRODUCTION_BY_UNCOUPLING_PROTEINS | 1.9164529 | 0.01284797 | 0.01260611 | 2.04819 | 0 | 0.0063877 |
| REACTOME_RNA_POLYMERASE_I_PROMOTER_ESCAPE | 1.8431549 | 0.0043384 | 0.01874117 | 1.495363 | 0.09936575 | 0.14344549 |
| REACTOME_RNA_POLYMERASE_I_TRANSCRIPTION | 1.8778145 | 0.00218341 | 0.01535967 | 1.4002613 | 0.12989691 | 0.21561971 |
| REACTOME_RNA_POLYMERASE_I_TRANSCRIPTION_INITIATION | 2.068652 | 0.00436681 | 0.00497651 | 1.7154548 | 0.01788909 | 0.04764653 |

| | | | | | | |
|---|---|---|---|---|---|---|
| REACTOME_RNA_POLYMERASE_I_TRANSCRIPTION_TERMINATION | 1.9953035 | 0.00223214 | 0.00723592 | 2.0227854 | 0 | 0.00677728 |
| REACTOME_RNA_POLYMERASE_II_PRE_TRANSCRIPTION_EVENTS | 2.3488002 | 0 | 0.00267704 | 1.3820148 | 0.10836502 | 0.22750261 |
| REACTOME_RNA_POLYMERASE_II_TRANSCRIBES_SNRNA_GENES | 2.239864 | 0 | 0.00152622 | 1.4562498 | 0.07254902 | 0.17383748 |
| REACTOME_RNA_POLYMERASE_II_TRANSCRIPTION_ELONGATION | 2.3306353 | 0 | 0.00133852 | 1.6016752 | 0.03838772 | 0.08972499 |
| REACTOME_RNA_POLYMERASE_II_TRANSCRIPTION_PRE_INITIATION_AND_PROMOTER_OPENING | 2.1316564 | 0 | 0.00393958 | 1.6443615 | 0.02348337 | 0.07167759 |
| REACTOME_RNA_POLYMERASE_II_TRANSCRIPTION_TERMINATION | 2.3057413 | 0 | 9.07E-04 | 1.651072 | 0.04230769 | 0.06881595 |
| REACTOME_RNA_POLYMERASE_III_CHAIN_ELONGATION | 2.016082 | 0 | 0.00609304 | 1.9593205 | 0.00406504 | 0.00871907 |
| REACTOME_RNA_POLYMERASE_III_TRANSCRIPTION | 2.0885127 | 0 | 0.00443711 | 1.7652147 | 0.01147228 | 0.03551478 |
| REACTOME_RNA_POLYMERASE_III_TRANSCRIPTION_INITIATION_FROM_TYPE_1_PROMOTER | 2.0441093 | 0.00234192 | 0.0055129 | 1.9414744 | 0.002 | 0.00990661 |
| REACTOME_RNA_POLYMERASE_III_TRANSCRIPTION_INITIATION_FROM_TYPE_3_PROMOTER | 2.1432269 | 0 | 0.00353843 | 1.7855624 | 0.00803213 | 0.03262036 |
| REACTOME_RNA_POLYMERASE_III_TRANSCRIPTION_TERMINATION | 2.0291727 | 0 | 0.00549673 | 1.7599305 | 0.01394422 | 0.03642537 |
| REACTOME_RRNA_MODIFICATION_IN_THE_NUCLEUS_AND_CYTOSOL | 2.0005398 | 0 | 0.00703263 | 1.9754075 | 0.00194553 | 0.00834785 |
| REACTOME_RRNA_PROCESSING | 2.199985 | 0 | 0.00190777 | 2.162135 | 0 | 0.00310369 |
| REACTOME_RRNA_PROCESSING_IN_THE_NUCLEUS_AND_CYTOSOL | 2.190382 | 0 | 0.00201543 | 2.1477134 | 0 | 0.00344542 |
| REACTOME_RUNX1_REGULATES_TRANSCRIPTION_OF_GENES_INVOLVED_IN_DIFFERENTIATION_OF_HSCS | 1.8083436 | 0.01268499 | 0.02341706 | 1.7346545 | 0.04393305 | 0.04232642 |
| REACTOME_S_PHASE | 1.9222745 | 0.0022779 | 0.01219908 | 1.9254946 | 0.01535509 | 0.0110913 |
| REACTOME_SCF_SKP2_MEDIATED_DEGRADATION_OF_P27_P21 | 1.9556371 | 0 | 0.00959038 | 1.9685636 | 0.00397614 | 0.00854182 |
| REACTOME_SELENOAMINO_ACID_METABOLISM | 2.0290227 | 0.00201613 | 0.00541344 | 2.063341 | 0 | 0.00714493 |
| REACTOME_SIGNALING_BY_FGFR2_IIIA_TM | 1.8843486 | 0 | 0.01499909 | 1.8305104 | 0.00399202 | 0.02410317 |
| REACTOME_SIGNALING_BY_NOTCH | 1.5436913 | 0.06315789 | 0.10377374 | 1.4272561 | 0.09090909 | 0.19442068 |
| REACTOME_SIGNALING_BY_NOTCH4 | 1.8296386 | 0.01290323 | 0.02023964 | 1.8343575 | 0.01359223 | 0.02351731 |
| REACTOME_SIGNALING_BY_ROBO_RECEPTORS | 2.3095887 | 0.00207469 | 0.00105789 | 2.2294707 | 0 | 0.00322431 |
| REACTOME_SRP_DEPENDENT_COTRANSLATIONAL_PROTEIN_TARGETING_TO_MEMBRANE | 1.9144826 | 0.00199601 | 0.01278106 | 2.0418425 | 0 | 0.00643421 |
| REACTOME_STABILIZATION_OF_P53 | 1.9270846 | 0 | 0.01190125 | 1.9845948 | 0 | 0.00775834 |
| REACTOME_SUMOYLATION_OF_DNA_REPLICATION_PROTEINS | 1.9026752 | 0.00444445 | 0.01366486 | 1.397222 | 0.15140186 | 0.216854 |
| REACTOME_SWITCHING_OF_ORIGINS_TO_A_POST_REPLICATIVE_STATE | 1.9500824 | 0 | 0.01009137 | 2.1171749 | 0 | 0.00462346 |
| REACTOME_SYNTHESIS_OF_ACTIVE_UBIQUITIN:_ROLES_OF_E1_AND_E2_ENZYMES | 1.8224051 | 0 | 0.02137468 | 1.6047342 | 0.03769841 | 0.08882769 |
| REACTOME_TELOMERE_C_STRAND_LAGGING_STRAND_SYNTHESIS | 1.5864693 | 0.04328018 | 0.08613811 | 1.826259 | 0.00976563 | 0.02469887 |
| REACTOME_TELOMERE_MAINTENANCE | 1.7377082 | 0.00455581 | 0.03751328 | 1.7577046 | 0.02505219 | 0.03674061 |
| REACTOME_TERMINATION_OF_TRANSLESION_DNA_SYNTHESIS | 1.8017545 | 0.03131991 | 0.02384412 | 1.7116531 | 0.01919386 | 0.04857391 |
| REACTOME_THE_CITRIC_ACID_TCA_CYCLE_AND_RESPIRATORY_ELECTRON_TRANSPORT | 1.9379157 | 0.0155902 | 0.0109947 | 2.1370785 | 0 | 0.00332276 |
| REACTOME_THE_ROLE_OF_GTSE1_IN_G2_M_PROGRESSION_AFTER_G2_CHECKPOINT | 1.9745617 | 0 | 0.00861197 | 2.0885015 | 0 | 0.00583854 |
| REACTOME_TNFR2_NON_CANONICAL_NF_KB_PATHWAY | 1.4032557 | 0.12549801 | 0.18304141 | 1.8214812 | 0.01619433 | 0.02565606 |
| REACTOME_TP53_REGULATES_METABOLIC_GENES | 1.686296 | 0.04564316 | 0.05072368 | 1.6824156 | 0.02514507 | 0.05964736 |
| REACTOME_TP53_REGULATES_TRANSCRIPTION_OF_DNA_REPAIR_GENES | 2.2151768 | 0.00225225 | 0.00199453 | 1.5824646 | 0.03932584 | 0.09688747 |
| REACTOME_TRANSCRIPTION_COUPLED_NUCLEOTIDE_EXCISION_REPAIR_TC_NER | 2.0399885 | 0.00455581 | 0.00538842 | 2.0384967 | 0 | 0.0060144 |
| REACTOME_TRANSCRIPTION_OF_THE_HIV_GENOME | 2.2898886 | 0 | 9.04E-04 | 1.5045493 | 0.06641366 | 0.14081663 |
| REACTOME_TRANSCRIPTIONAL_REGULATION_BY_RUNX1 | 1.6577017 | 0.04237288 | 0.05837716 | 1.3591067 | 0.12804878 | 0.24670947 |
| REACTOME_TRANSCRIPTIONAL_REGULATION_BY_RUNX2 | 1.4940515 | 0.1147541 | 0.1281516 | 1.6971443 | 0.02321083 | 0.05404452 |
| REACTOME_TRANSCRIPTIONAL_REGULATION_BY_RUNX3 | 2.0440857 | 0.00210971 | 0.00541445 | 1.731981 | 0.01937985 | 0.04265963 |
| REACTOME_TRANSCRIPTIONAL_REGULATION_BY_TP53 | 2.1288748 | 0 | 0.00370308 | 1.5226176 | 0.04112149 | 0.13067484 |
| REACTOME_TRANSLATION | 2.2100782 | 0 | 0.0019223 | 2.2100973 | 0 | 0.0017895 |
| REACTOME_TRANSLESION_SYNTHESIS_BY_POLH | 1.6726143 | 0.03603604 | 0.05407743 | 1.6739649 | 0.03 | 0.06196893 |
| REACTOME_TRANSLESION_SYNTHESIS_BY_POLK | 1.6166819 | 0.06436782 | 0.07381504 | 1.504542 | 0.1027668 | 0.14015241 |
| REACTOME_TRANSLESION_SYNTHESIS_BY_Y_FAMILY_DNA_POLYMERASES_BYPASSES_LESIONS_ON_DNA_TEMPLATE | 1.8833784 | 0.01342282 | 0.01498709 | 1.66822 | 0.0270793 | 0.06299011 |
| REACTOME_TRANSPORT_OF_MATURE_TRANSCRIPT_TO_CYTOPLASM | 2.340663 | 0 | 0.0017847 | 1.4972394 | 0.08070866 | 0.14256117 |
| REACTOME_TRISTETRAPROLIN_TTP_ZFP36_BINDS_AND_DESTABILIZES_MRNA | 1.6072316 | 0.04347826 | 0.07738736 | 1.6272004 | 0.04457364 | 0.07930689 |
| REACTOME_TRNA_AMINOACYLATION | 1.8575145 | 0.00223714 | 0.01728679 | 1.9976845 | 0 | 0.00715609 |
| REACTOME_TRNA_MODIFICATION_IN_THE_NUCLEUS_AND_CYTOSOL | 1.852859 | 0.00442478 | 0.01780637 | 1.7998266 | 0.00772201 | 0.0306771 |
| REACTOME_TRNA_PROCESSING | 2.1589043 | 0 | 0.0033088 | 1.9569283 | 0.00190114 | 0.0088311 |
| REACTOME_TRNA_PROCESSING_IN_THE_NUCLEUS | 2.2465785 | 0 | 0.00153048 | 1.7821378 | 0.01388889 | 0.03280213 |
| REACTOME_UB_SPECIFIC_PROCESSING_PROTEASES | 1.9393744 | 0.0021692 | 0.01089921 | 1.4543052 | 0.08171206 | 0.17474441 |
| REACTOME_UCH_PROTEINASES | 2.1233668 | 0 | 0.00388509 | 1.9216232 | 0.00403226 | 0.01126695 |
| REACTOME_UNFOLDED_PROTEIN_RESPONSE_UPR | 1.4439588 | 0.13179916 | 0.15693074 | 1.5135304 | 0.05870021 | 0.13586117 |
| REACTOME_VIF_MEDIATED_DEGRADATION_OF_APOBEC3G | 1.806462 | 0.00218818 | 0.02347024 | 1.9886737 | 0 | 0.00757899 |
| REACTOME_VIRAL_MESSENGER_RNA_SYNTHESIS | 2.113393 | 0 | 0.00416071 | 1.8369535 | 0.00934579 | 0.02316331 |
